# Combination of FTO and BTK inhibitors synergistically suppresses the malignancy of breast cancer cells

**DOI:** 10.7150/ijbs.117999

**Published:** 2025-10-27

**Authors:** Abdulaziz Ahmed A. Saad, Lichen Ge, Haoran Wang, Yan Xia, Jianing Li, Shiyao Qiao, Cheng Yi, Xiansong Wang, Zhaotong Wang, Dan Zhou, Hongsheng Wang

**Affiliations:** 1 Guangdong Provincial Key Laboratory of Chiral Molecule and Drug Discovery, The State Key Laboratory of Anti-Infective Drug Discovery and Development, School of Pharmaceutical Sciences, Sun Yat-Sen University, Guangzhou 510006, China.; 2 Department of Laboratory Medicine, Third Affiliated Hospital of Sun Yat-sen University, Guangzhou 510630, China.; 3 Science and Technology Innovation Center, Guangzhou University of Chinese Medicine, Guangzhou, 510006, China.; 4 Department of Breast Surgery, Southern University of Science and Technology Affiliated Foshan Hospital (The First People's Hospital of Foshan), Foshan 528000, Guangdong Province, China.

**Keywords:** FTO inhibitor FB23, Ibrutinib, c-Myc, E2F1, Breast cancer

## Abstract

Despite significant progress in breast cancer treatment, more effective methods for its clinical management are still needed. Our data identified that fat mass and obesity-associated protein (FTO), an *N*^6^-methyladenosine (m^6^A) demethylase, is highly expressed in breast cancer and promotes tumorigenesis. Inhibiting FTO can suppress the proliferation and metastasis of breast cancer, while its efficacy needs to be further improved. Through screening with 27 clinically approved targeted therapy drugs, we discovered that ibrutinib, a BTK inhibitor, shows the highest cell death rate and lowest combination index (CI). This combination demonstrates a potent synergistic effect in the malignancy of breast cancer and its lung metastasis. RNA-seq showed that the oncogenic pathways regulated by c-Myc and E2F1 were among the most down-regulated in cells treated with FTO inhibitor and ibrutinib. Furthermore, this combination decreases the expression of both c-Myc and E2F1. Contrarily, overexpressing c-Myc and E2F1 counteracts this antitumor effectiveness. Mechanistically, this combination inhibits c-Myc and E2F1 expression by increasing m^6^A modification of their mRNAs and reducing their mRNA stability. In mouse models of cancer, combining FTO knockdown with ibrutinib markedly suppressed tumor growth, decreased metastasis, and improved survival. Collectively, the combined inhibition of FTO and BTK exhibited substantial synergistic anticancer effects in breast cancer. Our findings advocate for the evaluation of this combination in clinical trials.

## Introduction

Despite ongoing medical advancements, breast cancer continues to be the second leading cause of death among women worldwide 1, 2. In 2020, there were around 2.3 million new breast cancer cases and approximately 685,000 deaths worldwide 3. Breast cancer is clinically categorized into three primary subtypes based on the status of hormone receptors and human epidermal growth factor receptor 2 (HER2): luminal, which is estrogen receptor (ER)-positive and progesterone receptor (PR)-positive and further divided into luminal A and B; HER2-positive; and triple-negative breast cancer (TNBC) 4. Breast cancer's high heterogeneity primarily results in mortality due to chemo-resistance and metastasis. This emphasizes the urgent need for innovative treatments tailored to combat this aggressive cancer 5, 6. Targeted drug therapies focus on proteins that facilitate the growth and spread of breast cancer cells 7. Exploring novel therapeutic strategies, such as combination treatments, is vital and promising for enhancing outcomes for those affected by this challenging disease.

*N*^6^-methyladenosine (m^6^A) is recognized as the predominant modification within messenger RNA (mRNA), playing a pivotal role in the regulation of RNA processing, stability, and translation 8. The fat mass and obesity-associated (FTO) protein, the first RNA m^6^A demethylase discovered, plays a significant oncogenic role in various cancers 9, notably breast cancer 9-11. These findings highlight FTO as a potential target for cancer therapies. Hence, FTO inhibitors, namely FB23, which directly bind and selectively inhibit FTO's m^6^A demethylase activity, offer promising prospects in cancer treatment 9, 12. This targeted approach translates to impressive results, demonstrating significantly stronger inhibitory effects on FTO enzymatic activity reducing cancer cell viability 12, 13. Notably, FB23 exhibited almost 140-fold stronger activity than meclofenamic acid (MA) in inhibiting FTO enzymatic activity in acute myeloid leukemia (AML) 9, 13. Moreover, the combination of FTO inhibitors with other targeted therapies has demonstrated enhanced therapeutic efficacy. For instance, the co-administration of rhein and nilotinib has shown potential in improving treatment outcomes in AML by enhancing cancer cell sensitivity 14. Similarly, the FB23-everolimus combination effectively suppresses the growth of pancreatic neuroendocrine tumors 15. In breast cancer, dual inhibition of FTO and the PDK1-AKT signaling pathway using FB23 and BX-912 has been shown to significantly reduce tumor progression 16. This emerging approach, targeting multiple pathways simultaneously, holds the potential as a valuable and powerful therapeutic strategy. However, current therapeutic strategies predominantly focus on regulating the tumor microenvironment, neglecting the full potential of FTO inhibitors in combinatorial approaches. More research is crucial to explore the therapeutic potential of combining FTO inhibitors with other targets for a more comprehensive and potentially effective treatment strategy.

Enhancing treatment outcomes for breast cancer often requires combination therapy, including multiple targeted agents. This approach constitutes the standard of care and underpins both surgical de-escalation in the breast and risk-adapted post-neoadjuvant approaches 2, 4. Moreover, while the "one drug, one target" paradigm offers notable specificity and potency in cancer treatment, single-target drugs face substantial challenges, including issues with drug resistance, limited pharmacokinetic profiles, and suboptimal patient adherence 17. Consequently, recognizing the multifaceted nature of cancer, the concurrent inhibition of two targets by a combination of drug entities emerges as a valuable and effective therapeutic approach against cancer 18. Ibrutinib, an irreversible Bruton's tyrosine kinase (BTK) inhibitor, is approved for the treatment of various B-cell malignancies 19. Ibrutinib, a promising anticancer therapy, garners interest in its potential efficacy not only in B-cell malignancies but also in solid tumors, notably breast cancer 19-21. Preclinical studies consistently validate ibrutinib's effectiveness in breast cancer cell lines and animal models, highlighting its therapeutic potential 22. Furthermore, combined inhibition of BTK with other targets demonstrates synergistic effects, providing a strategy to overcome drug resistance, lower therapeutic doses, and mitigate side effects 23.

Our data showed that FTO is highly expressed in breast cancer and enhances tumorigenic activity. FTO inhibitors can suppress the proliferation and metastasis of breast cancer, while its efficacy is not satisfactory and needs to be further improved. We further assess the potential synergistic antitumor effects of combining FTO inhibitors and other clinically approved targeted therapy drugs for breast cancer. Our results clearly demonstrate that the combination of FB23 and ibrutinib acts synergistically, effectively suppressing the growth of breast cancer and lung metastatic breast cancer (LMBC) cells both *in vitro* and *in vivo*. Mechanistically, this synergy involves the downregulation of key oncogenic transcripts, c-Myc and E2F1, leading to suppressed tumor growth. These promising findings strongly advocate for further exploration of the FB23 and ibrutinib combination as a compelling therapeutic strategy for breast cancer patients.

## Materials and Methods

### Drugs and reagents

Ibrutinib and FB23 were provided by Targetmol (Boston, MA); both were dissolved in DMSO (Sigma Aldrich, Darmstadt, Germany) at 10 µM and stored at -80°C. All oncology drugs used are listed in Table S1.

### Cell culture

Human breast cancer cell lines, including MDA-MB-231 and BT-549, as well as the normal breast epithelial cell line MCF10A and human kidney epithelial cell line 293T were purchased from the American Type Culture Collection (ATCC, Manassas, VA) and maintained by our laboratory. Cell lines were authenticated by short tandem repeat (STR) profiling (Shanghai Biowing Applied Biotechnology Co., Ltd) and tested negative for mycoplasma using the Myco-Blue Mycoplasma Detector (Vazyme) prior to the experiments. The cells cultured in Dulbecco's Modified Eagle Medium (DMEM, Gibco, Carlsbad, CA, USA) supplemented with 10% fetal bovine serum (FBS) and 1% penicillin-streptomycin (Gibco). MCF10A cells were cultured in DMEM/F-12 (Gibco, Carlsbad, CA, USA) with 10% FBS, 0.5 µg/mL hydrocortisone, 10 µg/mL insulin, 20 ng/mL EGF, and antibiotics. The cell lines were maintained in a 37°C, 5% CO_2_ humidified incubator.

### Establishment of breast cancer cell models with lung metastasis (LM)

Our laboratory previously described the process for creating LM models from breast cancer cells 24. Briefly, we first harvested the initial generation of LM cells from the lungs of four-week-old immunodeficient mice, following the injection of 2×10^5^ viable MDA-MB-231 or BT-549 cells into the lateral tail vein in a volume of 0.1 mL. After eight weeks, we isolated the cells that had metastasized to the lungs. Then breast cancer cells metastasized to the lung were isolated, primarily cultured with medium, and named as MDA-MB-231^LMF1^, MDA-MB-231^LMF2^ and MDA-MB-231^LMF3^ or BT-549^LMF1^, BT-549^LMF2^ and BT-549^LMF3^ cells which were similarly generated from their corresponding parental cells. These cells were primarily cultured in a medium supplemented with 10% FBS and were designated as MDA-MB-231^LMF3^ or BT-549^LMF3^, highlighting their enhanced potential for lung metastasis.

### Cell viability assay

Cell viability was evaluated using a cell counting kit-8 (CCK-8). Cells were seeded at appropriate densities in 96-well plates using their respective growth medium and allowed to adhere overnight until they achieved 75%-80% confluence at the end of the assay for each cell line. Once the cells reached confluence, they were treated with or without drugs for the designated time. After treatment as indicated, 10 μL of CCK-8 reagent was added to each well containing 100 μL of medium and incubated at 37 °C for 2 h. Absorbance was measured at 450 nm using an automatic multi-hole spectrophotometer. Viability was calculated as the percentage of absorbance in treated wells compared to control.

### Cell migration and invasion assays

For the wound healing assay, 4×10^5^ cells were seeded in 6-well plates. Once reaching 90% confluency, the cell monolayer was carefully scratched using a sterile pipette tip to create a vertical wound. After three washes, the cells were cultured in an FBS-free medium, and the scratched area was measured in randomly chosen fields under a microscope at 0 and 24 h. The percentage of the wounded area was calculated as follows: (mean remaining breadth/mean wounded breadth) ×100%.

The transwell assay was conducted using CytoSelect™ 24-well cell invasion assay kits coated with matrigel (BD Bioscience, Bedford, MA). In the upper chamber, 1×10^5^ cells in 200 μL of culture medium (supplemented with 0.1% FBS) were added, while the lower chamber was supplemented with 600 μL of medium containing 10% FBS. After incubation, the cells that had invaded the lower chamber were fixed and stained. Finally, the number of invaded cells was counted in 5 random fields under an upright microscope.

### Clonogenic assay

Cells were distributed into 6-well plates, with each well containing 5×10^2^ cells, and left to adhere overnight in the culture medium. Cells were then cultured for 12-14 days in complete medium supplemented with either the drug(s) alone, drug combinations, the target gene, or vehicle, as per the experimental design. The growth media was refreshed at regular intervals of 48 h. After the specified incubation period, the cells were fixed with 0.4% buffered paraformaldehyde for 15 min and then stained with 0.5% crystal violet for 15 min. Then, colony numbers were counted using ImageJ software and compared for each passage.

### RIP-RT-qPCR

The RNA immunoprecipitation real-time qPCR (RIP-RT-qPCR) procedure followed the methods outlined in our previous study 25. In brief, 10 cm cell plates were washed with cold PBS and then lysed using 400 μL of IP lysis buffer on ice for 30 min. The clear lysate was collected after centrifugation at 12,000 g for 10 min. Subsequently, either 4 uL of targeted antibodies or IgG was incubated with Protein G Magnetic beads in 1×Reaction buffer (composed of 150 mM NaCl, 10 mM Tris-HCl at pH 7.5, and 0.1% NP-40 in nuclease-free H_2_O) at 4 °C for 3 h. Following this, there was incubation with 200 μL of extracted RNA at 4°C for 3 h. Proteinase K was then added to digest proteins in the immunoprecipitated RNA-protein complex, which was isolated using Trizol (Invitrogen, Carlsbad, CA, USA) for RNA extraction, followed by ethanol precipitation. Subsequently, the isolated RNAs of interest underwent reverse transcription and quantification through qPCR.

### mRNA stability

To assess RNA stability in MDA-MB-231 and BT-549 cells, we achieved cellular stability by treating the cells with actinomycin D (Act-D, Sigma, USA) at a concentration of 5 μg/mL. Subsequently, cells were collected at specified time intervals, and RNA extraction was performed for Real-time PCR analysis. c-Myc and E2F1 mRNA were determined by calculating the natural logarithm of 2 divided by the slope, with GAPDH used as the reference gene for normalization. The RNA degradation rate (*K*_decay_) was estimated using the following equation 26 (*ln (C/C_0_) = -K_decay_ t)* In this equation, *C_0_​* represents the concentration of mRNA at time zero, t denotes the duration of transcription inhibition, and *C* is the mRNA concentration at time t. Therefore, *K*_decay_ can be derived from the exponential decay fitting of the ratio *C*/*C_0_​* versus time t. The half-life (t*_1/2_*) is defined as the time at which the ratio *C*/*C_0_* equals 50%, or 1/2. This can be expressed mathematically as (*ln (1/2) = -K_decay_ t*_1/2_) Rearranging this equation yields the mRNA half-life (*t_1/2_ = ln2/K_decay_*).

### Western blotting

The western blotting technique was conducted to detect the expression of a gene in samples obtained after drug treatments. Briefly, protein samples were extracted using radioimmunoprecipitation assay (RIPA) buffer (Beyotime Biotechnology) supplemented with 1% phenylmethylsulfonyl fluoride (PMSF) as a protease inhibitor. Loading buffer (5×SDS, Beyotime Biotechnology) was then added to each protein sample at a 4:1 ratio. The samples were subsequently denatured by heating at 100 °C for 20 min. Then, samples were allowed to cool and stored at -20°C for long-term preservation. Protein samples were loaded for gel electrophoresis in 10% sodium dodecyl sulfate-polyacrylamide. After electrophoresis, proteins were transferred to prewetted polyvinylidene fluoride (PVDF) membranes (Millipore). The non-specific signals of PVDF membranes were then blocked in blocking buffer 5% skim-milk powder dissolved in 1×PBST for one hour at room temperature with slight shaking. Subsequently, the PVDF membranes were then kept with specific primary antibodies for 12 h inside the refrigerator 4°C with slight shanking on the lab rotator. The bands of proteins were pictured using the Tanon-5200 imaging system (Tanon) after 2 h of incubation with second antibodies. The antibodies utilized in this study are detailed in Table S2.

### Flow cytometry analysis

Cells were subjected to apoptosis assays using the Annexin V-FITC/PI kit (Dojindo, Kumamoto, Japan) after the designated treatment period, following the manufacturer's instructions. Subsequently, a Coulter Epics XL Flow Cytometry System (Beckman-Coulter, Miami, USA) was performed. In brief, cells were initially seeded at a density of 5×10^5^ in 6-well plates and allowed to incubate overnight at 37°C. Subsequently, we exposed the cells to drugs for 48 h. After this incubation period, we collected the cells, stained them with Annexin V-FITC and propidium iodide (PI) (each at 5%) for 15 min at room temperature in the dark, and then resuspension in 250 μL of detection buffer, the cells were analyzed using flow cytometry analysis and apoptotic cell quantification was performed using FlowJo VX software (Tristar, CA, United States).

### *In vivo* mouse studies

All animal experiments were performed according to protocols approved by the Zhongshan School of Medicine Policy on the Care and Use of Laboratory Animals. The *in vivo* experimental protocols were approved by the Institutional Animal Care and Use Committee. These *in vivo* studies encompassed both xenograft and metastasis models.

### Xenograft models

To assess the efficacy of combined FTO inhibition and ibrutinib therapy in tumor reduction, 5×10^6^ MDA-MB-231 cells, either shNC or FTO knockdown (shFTO), were subcutaneously injected into the mammary fat pads of female mice. Then the mice were divided into four groups: 1) mice injected with shNC cells and treated with DMSO; 2) mice injected with shFTO cells and treated with DMSO; 3) mice injected with shNC cells and treated with ibrutinib (10 mg kg^-1^, injected intraperitoneally daily 27); 4) mice injected with shFTO cells and treated with ibrutinib (10 mg kg^-1^, injected intraperitoneally daily). The treatment lasted for 23 days, with body weight and tumor size measured daily, starting when their tumor volumes reached approximately 100 mm³. After this period, the mice were euthanized, and the tumors were excised, weighed, and analyzed for the expression of Ki67, cleaved caspase 3, FTO, c-Myc, and E2F1.

### Metastasis models

First, to assess the impact of combining FTO inhibition and ibrutinib on reducing the metastatic potential of LMBC, we used the 5×10^6^ MDA-MB-231^LMF3^ shNC or shFTO cell line that were injected into the lateral tail vein of female mice, which were then divided into 2 groups: 1) mice injected with MDA-MB-231^LMF3^ cells (shNC) treated with DMSO; 2) mice injected with shFTO MDA-MB-231^LMF3^ cells treated with ibrutinib (10 mg kg^-1^, injected intraperitoneally daily). The treatment lasted for 4 weeks, starting two weeks after the cell injections. After this period, the mice were sacrificed, and their lungs were isolated and analyzed for the presence of metastatic tumors and histological analysis.

Second, to evaluate the survival impact of combining FTO inhibition and ibrutinib, we performed a tail-vein metastasis assay by injecting 5×10^6^ MDA-MB-231^LMF3^ cells (shNC or shFTO) into the lateral tail vein of mice. Treatment was started after two weeks. The mice were divided into 4 groups: 1) mice injected with shNC cells and treated with DMSO; 2) mice injected with shFTO cells and treated with DMSO; 3) mice injected with shNC cells and treated with ibrutinib (10 mg kg^-1^, injected intraperitoneally daily); 4) mice injected with shFTO cells and treated with ibrutinib (10 mg kg^-1^, injected intraperitoneally daily). The treatment regimen was maintained until the first mice met the criteria for humane endpoints. The duration of survival was computed from the day of cell inoculation (designated as day 0) until the day of the animal's demise. For each group, survival trends were visualized using Kaplan-Meier survival plots.

### Database analysis

We collected RNA-seq data from The Cancer Genome Atlas (TCGA) breast cancer dataset and the GEO datasets GSE9014 and GSE193103. After removing batch effects, we merged the datasets for subsequent analysis, including gene expression quantification and survival analysis. The GEPIA 2.0 database was used to validate gene expression correlations 28. Differential FTO gene expression between tumor and normal tissues was assessed using TNMplot 29.

### Statistical analysis

The computation of means and standard deviations was performed using the software GraphPad Prism 9.0 (GraphPad Software Inc.). Statistical comparisons between two groups were performed using Student's t-test, unless otherwise specified. For comparisons involving multiple groups, one-way ANOVA was conducted, followed by either Tukey's or Dunnett's post hoc multiple comparisons test, as appropriate. *P* value of < 0.05 was statistically significant. The drug combination index (CI) quantifies the effect of two drugs on cell proliferation. It's calculated as CI = (V_combination_​​) /(V_ibrutinib_​×V_FB23_​), where D (%) is the cell survival rate, V_combination_​ is the ratio of D in the drug combination group, and V_ibrutinib_​ and V_FB23_​ are the ratios of survival rate D in the groups where each drug is used individually. The CI value interpretation is as follows: CI < 1: synergistic effect, CI = 1: additive effect, and CI > 1: antagonistic effect.

## Results

### FTO serves as a potential target for breast cancer therapy

**Figure 1:**
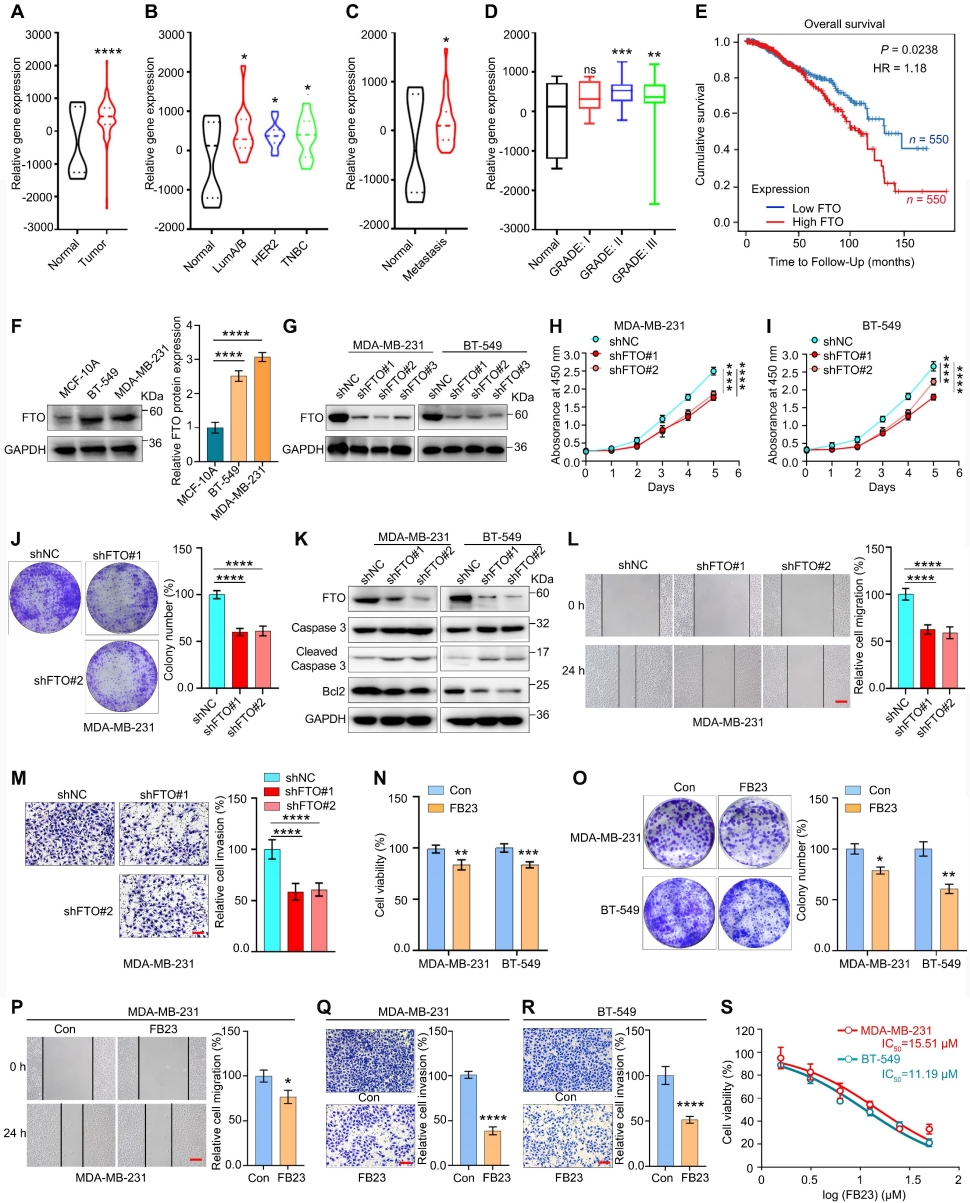
** FTO serves as a potential target for breast cancer therapy. (A)** Relative FTO mRNA expression levels in normal breast and breast tumor tissues, based on data available from the GSE9014 database. **(B)** Relative FTO mRNA expression levels in normal breast and different molecular subtypes of breast tumor tissues, based on data available from the GSE9014 database. **(C)** Relative FTO mRNA expression levels in normal breast and metastatic breast tumor tissues, based on data from the GSE9014 and GSE193103 databases. **(D)** Relative FTO mRNA expression levels across normal breast and different stages of breast tumor tissues, based on data from the GSE9014 database. **(E)** Kaplan-Meier survival curves showing overall survival (OS) based on FTO mRNA expression in breast cancer patients from the TCGA database. **(F)** Protein expression levels of FTO in MDA-MB-231 and BT-549 breast cancer cell lines, and in the non-tumorigenic MCF-10A cell line. **(G)** Protein expression levels of FTO in MDA-MB-231 and BT-549 breast cancer cells with stable knockdown (KD) of FTO. **(H-I)** Effects of FTO KD on the proliferation of MDA-MB-231 (H) and BT-549 (I) cells, as measured by the CCK-8 assay. **(J)** Colony formation assays assessing the impact of FTO KD on MDA-MB-231 cell proliferation for 12 days. **(K)** Impact of FTO KD on protein expression levels of apoptosis markers—caspase 3, cleaved caspase 3, and Bcl2—in MDA-MB-231 and BT-549 cells. **(L)** Migration assays evaluating the effect of FTO KD on the migration of MDA-MB-231 cells. **(M)** Invasion assays evaluating the effect of FTO KD on the invasion of MDA-MB-231 cells. **(N)** Cell proliferation quantified using the CCK-8 assay to assess the effects of FB23 (2.5 µM) on MDA-MB-231 and BT-549 cell lines after 24 h treatment. **(O)** Colonization of MDA-MB-231 and BT-549 cells treated with FB23 (2.5 µM) for 12 days, compared to untreated control groups. **(P)** Migration ability of MDA-MB-231 cells treated with FB23 (2.5 µM) for 24 h, compared to untreated control groups. **(Q**-**R)** Invasion capacities of MDA-MB-231 (Q) and BT-549 (R) cells treated with FB23 (2.5 µM) for 48 h, compared to untreated control groups. **(S)** Evaluation of the viabilities of MDA-MB-231 and BT-549 cell lines treated with FB23 for 24 h using the CCK-8 assay. The IC_50_ values were determined. *P* values (*P < 0.05, **P < 0.01, ***P < 0.001, ****P < 0.0001) were calculated using either an unpaired, two-tailed Student's t-test or one-way ANOVA, followed by the Dunnett test. Data are presented as mean ± SD. Scale bar = 100 µm.

FTO has been reported to be upregulated in various tumors, and its elevated levels are independently associated with a decreased overall survival (OS) rate in various cancer patients 9. We first checked the expression of FTO in breast cancer. Analysis using the TNMplot database revealed pan-cancer upregulation of FTO expression in multiple tumors, including breast cancer (Fig. S1A). We then analyzed FTO expression using the GSE9014 dataset from the GEO database, which showed that FTO is upregulated in breast cancer tumor tissues compared to normal tissues (Fig. 1A). Subsequently, further subtype-specific analysis revealed significantly elevated FTO expression in the three main clinical subtypes of breast cancer, compared to normal tissue (Fig. 1B), which reflects the broader trend observed across breast cancer. Additionally, analyses of metastatic samples from GSE9014 and GSE193103 revealed a marked increase in FTO expression in metastatic lesions compared to normal tissue (Fig. 1C). Further analysis of FTO expression across different stages of breast cancer revealed a progressive increase in FTO expression with disease advancement (Fig. 1D). Survival analyses indicated that higher FTO expression correlates with lower OS in breast cancer patients (Fig. 1E), suggesting FTO upregulation worsens clinical outcomes.

In our analysis, FTO expression was significantly higher in the breast cancer cell lines MDA-MB-231 and BT-549 compared to the non-tumorigenic MCF-10A cell line (Fig. 1F). To explore the biological roles of FTO in breast cancer malignancy, we introduced shRNA-mediated FTO knockdown (KD) (shFTO#1, #2, and #3) into MDA-MB-231 and BT-549 cells (Fig. 1G and S1B-C). The KD efficiencies of shFTO#1 and shFTO#2 were optimal, supporting their use in subsequent experiments. In MDA-MB-231 and BT-549 cells with FTO KD (shFTO#1 and #2), cell proliferation was significantly reduced (Fig. 1H-I). Clonogenic assays also showed that FTO KD impaired colony formation in these cells (Fig. 1J and S1D). Furthermore, FTO KD increased caspase 3 cleavage and reduced Bcl2 expression, indicating enhanced apoptosis (Fig. 1K). In addition, FTO KD significantly inhibited cell migration and invasion in both MDA-MB-231 (Fig. 1L and M) and BT-549 (Fig. S1E and F) cell lines. These findings underscore the critical role of FTO in promoting breast cancer cell proliferation, migration, and invasion, demonstrate that its depletion suppresses these malignant behaviors.

Given the upregulation of FTO in breast cancer and its potential role in cancer initiation and progression, we examined the effects of FB23, a potent FTO inhibitor that exhibits 140-fold greater efficacy than MA in inhibiting FTO-mediated demethylation 12, on breast cancer cells. Our findings indicate that FB23 inhibited cell proliferation in MDA-MB-231 and BT-549 cells after treatment with FB23 (2.5 µM) for 24 h (Fig. 1N). Similarly, FB23 (2.5 µM) moderately inhibited colony formation in these cells after incubation with the drug for 12 days (Fig. 1O). Furthermore, FB23 (2.5 µM) inhibited cell migration (Fig. 1P and S1G) and invasiveness (Fig. 1Q and R) in MDA-MB-231 and BT-549 cells. However, its standalone activity was modest, not reaching optimal efficacy in breast cancer. Then, an analysis of the cytotoxicity of FB23 alone was conducted, and the results showed a half maximal inhibitory concentration (IC_50_) of 15.51 µM in MDA-MB-231 and 11.19 µM in BT-549 cells (Fig. 1S). Considering that many clinical breast cancer therapy drugs with IC_50_ values less than 1 µM, our data indicated that FB23 may not be sufficiently effective as a monotherapy for breast cancer. It underscores the importance of combining FB23 with other therapeutic targets to enhance its effectiveness against tumors.

### Dual inhibition of FTO and BTK synergistically targets breast cancer

**Figure 2:**
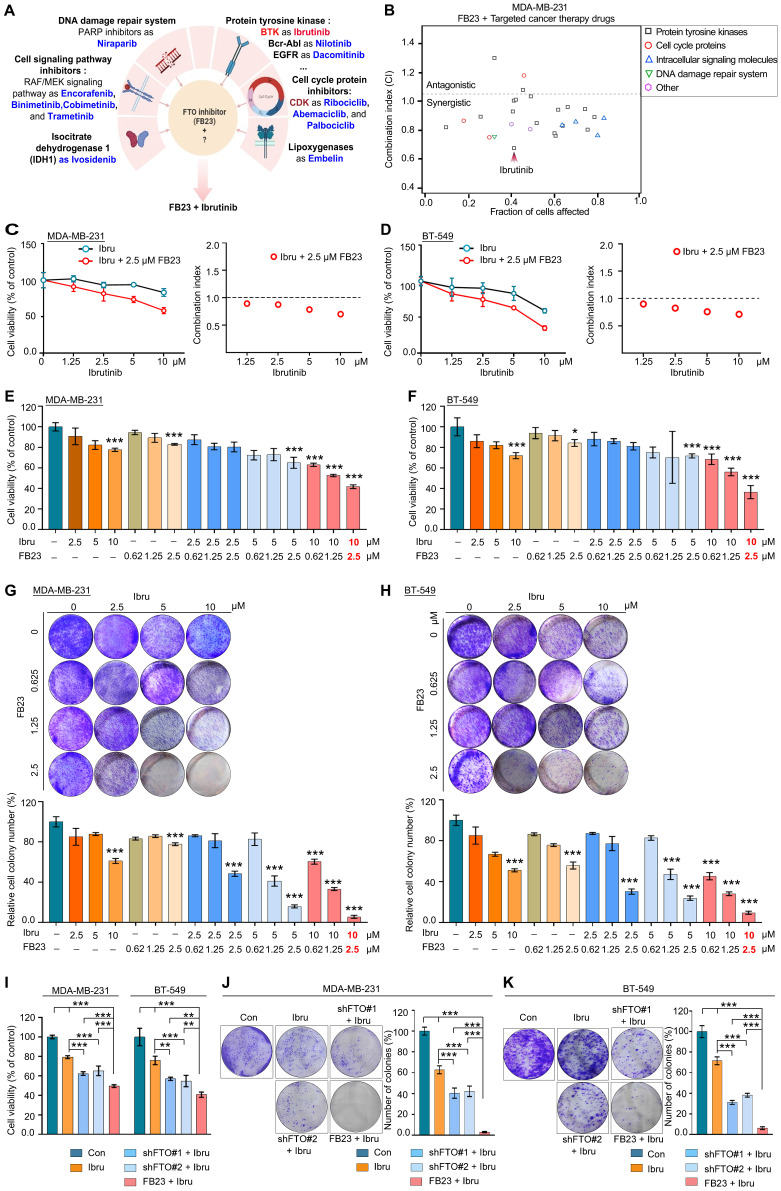
** Dual inhibition of FTO and BTK synergistically targets breast cancer. (A)** Schematic illustration of FB23 and predicted mechanisms of targeted cancer therapy drugs. **(B)** Screening for synergistic drug combinations with FTO inhibitors in MDA-MB-231 cells: Values below 1 indicate synergistic interactions, while values above 1 suggest antagonistic interactions. **(C**-**D)** Exploring synergistic effects of a combination of ibrutinib, a BTK inhibitor, and FB23, an FTO inhibitor, on MDA-MB-231 (C) and BT-549 (D) cells, including combination index analysis. **(E**-**F)** Evaluation of the viability of the combination of FB23 and ibrutinib in MDA-MB-231 (E) and BT-549 (F) cells; cells were incubated with escalating doses of ibrutinib (2.5, 5, 10 µM) and FB23 (0.62, 1.25, 2.5 µM) and their combinations for 24 h. Viability was assessed using the CCK-8 assay. **(G**-**H)** Colony formation assays evaluating the impact of FB23 and ibrutinib combination treatments on MDA-MB-231 (G) and BT-549 (H) cell proliferation. Cells were treated with escalating doses of ibrutinib (2.5, 5, 10 µM), FB23 (0.62, 1.25, 2.5 µM), or their combinations for 12 days. **(I)** Effects of FTO KD on the sensitivity of MDA-MB-231 and BT-549 cells to ibrutinib, as assessed by cell viability after 24 h. **(J-K)** Effects of FTO KD on the sensitivity of MDA-MB-231 (J) and BT-549 (K) cells to ibrutinib, as determined by colony formation assay. For E-H, indicated* P* values were determined by one-way ANOVA with Dunnett's test. For I-K, *P* values were determined by one-way ANOVA followed by Tukey's multiple comparisons test. **P < 0.01; ***P < 0.001, with data presented as mean ± SD.

The ability of FB23 to act synergistically with targeted drugs by inhibiting critical pathways involved in breast cancer progression makes it a promising candidate for use in combination therapies with targeted anticancer agents. We evaluated the antitumor potential of FB23 in combination with a selection of clinically approved targeted therapies. The 27 candidates included tyrosine kinase inhibitors (TKIs), cell cycle and signaling pathway inhibitors, DNA repair agents, and metabolic pathway targets such as lactate dehydrogenase and lipoxygenase, as detailed in Fig. 2A and Table S1. We assessed the viability of these cells and subsequently calculated the combination index (CI), which demonstrates that most clinical drugs targeting tyrosine kinases exhibited a synergistic effect when combined with FB23 (CI < 1 indicates synergy, CI > 1 indicates antagonism). Among the various combinations, the FB23 and ibrutinib combination demonstrated the highest rate of cell death and the lowest CI value (Fig. 2B), indicating the most potent synergistic effect on breast cancer cells.

Ibrutinib, the first inhibitor that irreversibly binds BTK, is approved for the treatment of various B-cell malignancies 19. Additionally, it has shown anticancer activity in solid tumors such as breast cancer 21, 22. We tested the gradient concentrations of FB23 and ibrutinib on MDA-MB-231 (Fig. 2C) and BT-549 (Fig. 2D) cells after a 24 h incubation, which showed the combination of 2.5 µM FB23 and 10 µM ibrutinib exhibited a strong synergy. We also evaluated the synergistic impact on cell proliferation of varying concentrations of FB23 (0.62, 1.25, 2.5 µM) and ibrutinib (2.5, 5, 10 µM), as well as their combinations, after 24 h. The results indicated that combining 2.5 µM of FB23 with 10 µM of ibrutinib significantly inhibited cell proliferation in MDA-MB-231 cells (Fig. 2E and S2A) and BT-549 cells (Fig. 2F and S2B). To further assess the synergistic effects, we utilized a colony formation assay across similar concentration ranges of FB23 and ibrutinib, treated for 12 days. Remarkably, the combination of 2.5 µM FB23 and 10 µM ibrutinib substantially suppressed colony growth in both MDA-MB-231 (Fig. 2G and S2C) and BT-549 (Fig. 2H and S2D) cell lines. These results underscore the substantial synergistic effect of 2.5 µM FB23 and 10 µM ibrutinib co-treatment, thereby suggesting their potential as a highly effective therapeutic strategy against breast cancer.

To validate that FTO KD sensitizes cells to ibrutinib, we treated FTO KD cells with ibrutinib and observed a significant decrease in cell viability upon FTO KD (Fig. 2I and S2E). This reduction in cell viability was further confirmed by the colony formation assay, where FTO KD cells formed significantly fewer colonies following ibrutinib treatment (Fig. 2J and K). These findings collectively suggest that FTO KD plays a critical role in regulating ibrutinib sensitivity in MDA-MB-231 and BT-549 breast cancer cells.

### FB23 and ibrutinib synergistically inhibit the malignancy of breast cancer cells

Observing the predominantly synergistic inhibition of cell proliferation in FB23 plus ibrutinib-amplified breast cancer cells, we assessed the duration effects of FB23 and ibrutinib, both as single agents and in combination.

**Figure 3:**
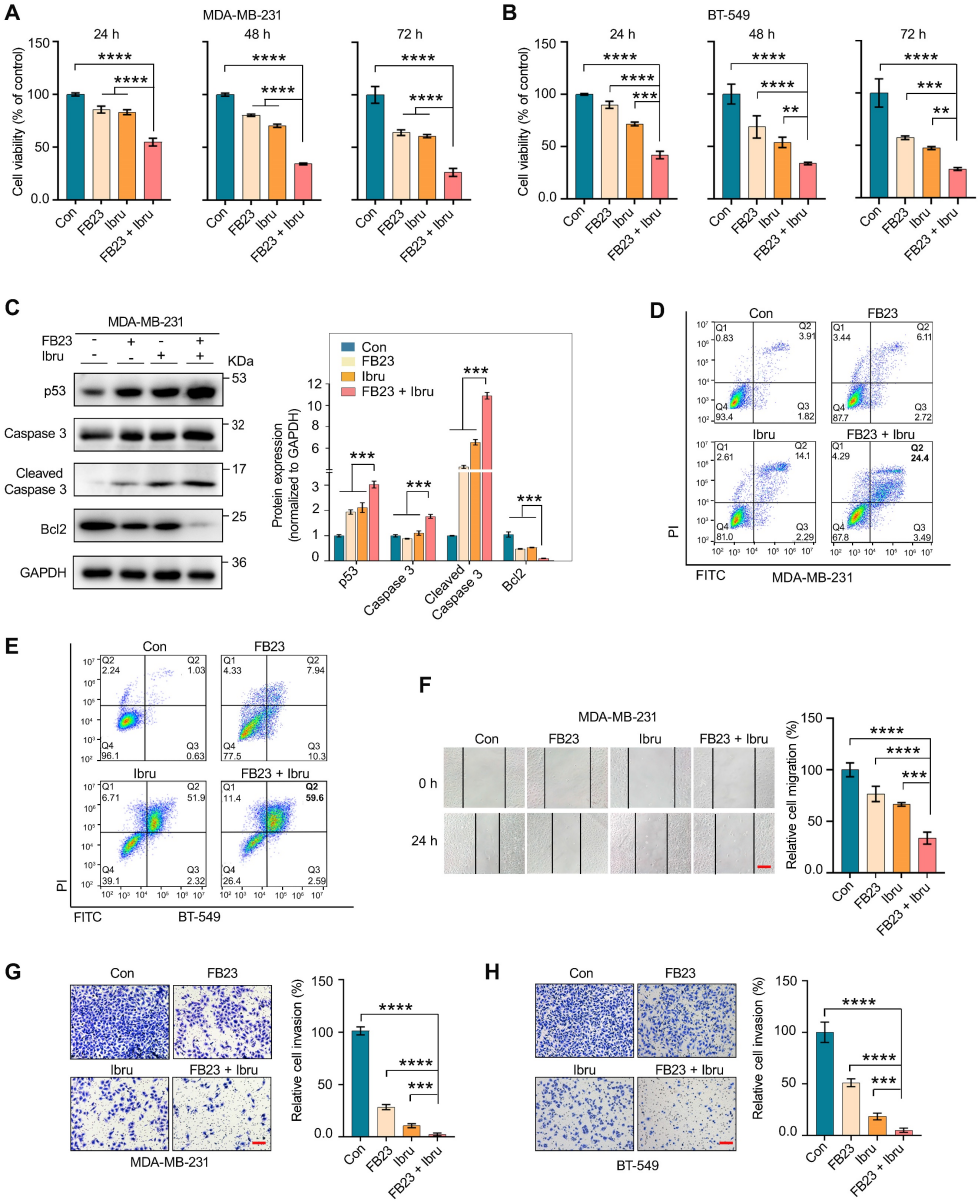
** FB23 and ibrutinib synergistically inhibit the malignancy of breast cancer cells. (A**-**B)** Cell viability of MDA-MB-231 (A) and BT-549 (B) cells treated with FB23 (2.5 µM), ibrutinib (10 µM), and their combination at various time points (24 h, 48 h, 72 h). Viability was assessed using the CCK-8 assay. **(C)** Protein expression levels of apoptosis markers—p53, caspase 3, cleaved caspase 3, and Bcl2—in MDA-MB-231 cells after 48 h treatment with FB23, ibrutinib, or their combination. **(D**-**E)** Flow cytometric evaluations of MDA-MB-231(D) and BT-549 (E) cells 48 h post-treatment with FB23 (2.5 µM), ibrutinib (10 µM), or their combination. **(F)** Migratory ability of MDA-MB-231 cells treated with FB23 (2.5 µM), ibrutinib (10 µM), or their combination for 24 h. **(G**-**H)** Invasion ability of MDA-MB-231 (G) and BT-549 (H) cells treated with FB23 (2.5 µM), ibrutinib (10 µM), or their combination for 48 h. Statistical analysis was performed using one-way ANOVA with Dunnett's test. *P* values (**P < 0.01, ***P < 0.001, ****P < 0.0001) are reported as mean ± SD. Scale bar = 100 µm

The results demonstrated that prolonged exposure to the combination treatment (at 24, 48, and 72 h) markedly enhanced the inhibition of cell proliferation compared with using each agent alone or with the control (Fig. 3A and B). Following a 48 h treatment with the drug combination resulted in a significant increase in caspase 3, cleaved caspase 3, and p53 protein expression levels, and a decrease in Bcl2 protein expression levels, compared to individual treatments or the control in MDA-MB-231 (Fig. 3C) and BT-549 (Fig. S3A) cells. Flow cytometry results revealed that the apoptotic rate of MDA-MB-231 (Fig. 3D) and BT-549 (Fig. 3E) cells significantly increased after 48 h treatment with a combination of FB23 and ibrutinib, as compared to FB23 or ibrutinib alone. These findings demonstrate that the combination of FB23 and ibrutinib synergistically suppresses the proliferation and increases the apoptotic potential of breast cancer cells.

We further assess the potential of combining FB23 and ibrutinib in curbing the migration and invasion of breast cancer cells. For migration abilities, the combination of FB23 (2.5 µM) and ibrutinib (10 µM) significantly reduced migration compared to FB23 alone, ibrutinib alone, or the control in MDA-MB-231 cells after 24 h of incubation (Fig. 3F). Similarly, in BT-549 cell assays, the combination treatment markedly reduced migration relative to single-agent treatments or the control after 24 h of incubation (Fig. S3B). For invasive abilities, the combination of FB23 (2.5 µM) and ibrutinib (10 µM) significantly decreased invasion compared to FB23, ibrutinib, or control in MDA-MB-231 cells (Fig. 3G). A similar reduction in invasion was observed in BT-549 cells with the combination treatment compared to either monotherapy or control (Fig. 3H). These results clearly demonstrate that the combined application of FB23 and ibrutinib effectively inhibits breast cancer, showcasing a synergistic antitumor effect.

### FB23 and ibrutinib synergistically inhibit malignancy of LMBC cells

**Figure 4:**
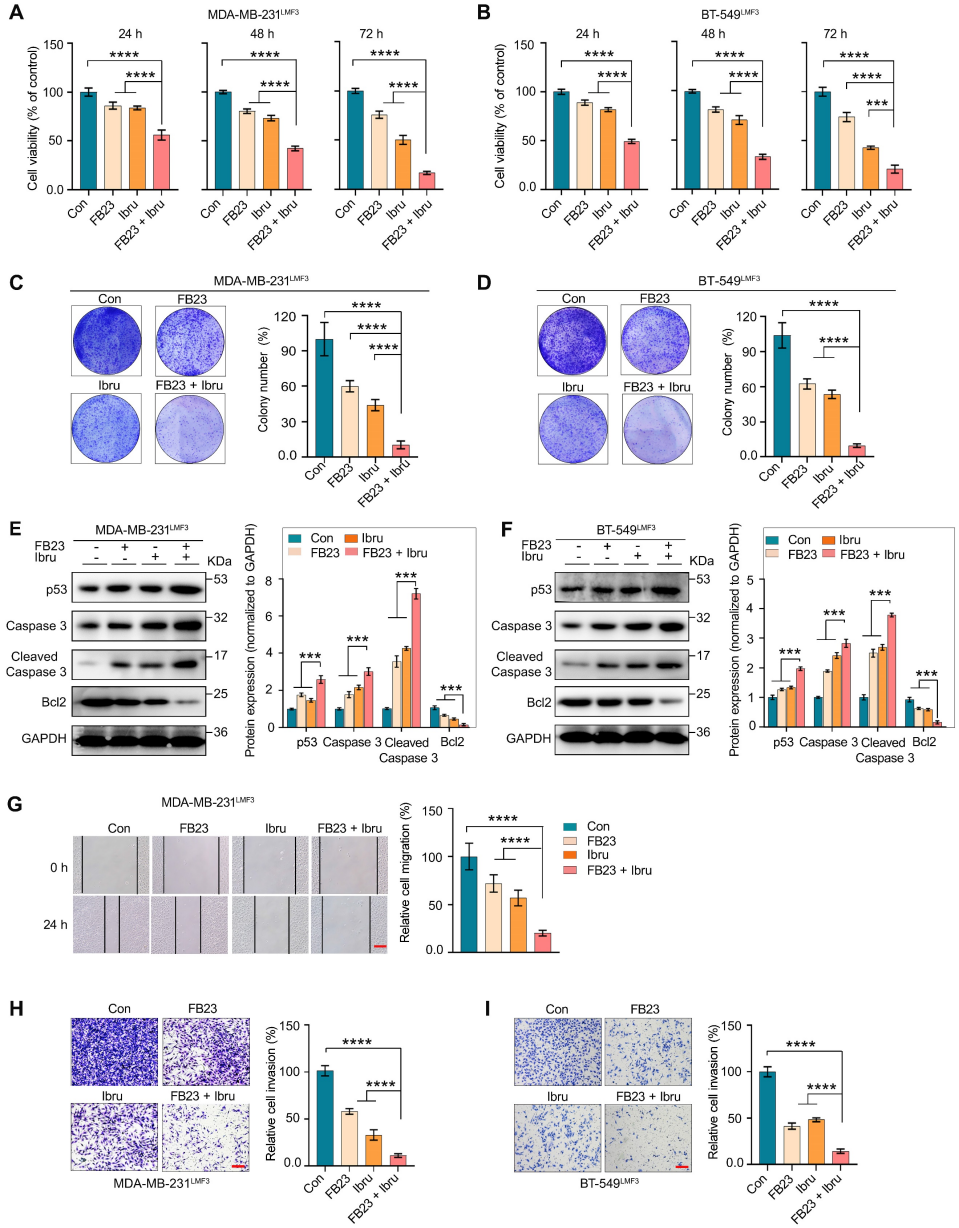
** FB23 and ibrutinib synergistically inhibit malignancy of LMBC cells. (A**-**B)** Assessment of cell viability in MDA-MB-231^LMF3^ (A) and BT-549^LMF3^ (B) cells treated with FB23 (2.5 µM), ibrutinib (10 µM), and their combination at 24, 48, and 72 h using the CCK-8 assay. **(C**-**D)** Colony formation assays of MDA-MB-231^LMF3^ (C) and BT-549^LMF3^ (D) cells treated with FB23 (2.5 µM), ibrutinib (10 µM), and their combination for 12 days. **(E**-**F)** Protein expression levels of apoptosis markers—p53, caspase 3, cleaved caspase 3, and Bcl2—in MDA-MB-231^LMF3^ (E) and BT-549^LMF3^ (F) cells 48 h after treatment with FB23 (2.5 µM), ibrutinib (10 µM), or their combination. **(G)** Migration assay of MDA-MB-231^LMF3^ cells treated for 24 h with FB23 (2.5 µM), ibrutinib (10 µM), and their combination. **(H**-**I)** Transwell invasion assay of MDA-MB-231^LMF3^ (H) and BT-549^LMF3^ (I) cells treated with FB23 (2.5 µM), ibrutinib (10 µM), or their combination. Experiments were terminated after seeding for 48 h. Statistical analysis was done using one-way ANOVA followed by Dunnett's multiple comparisons test. Data are presented as mean ± SD. *P* values (**P < 0.01, ***P < 0.001, ****P < 0.0001) are shown. Scale bar = 100 µm.

More than 60% of deaths related to breast cancer are associated with the presence of lung metastases 30. We further evaluated the effect of combination therapy on the malignancy of lung metastatic breast cancer cells, which were isolated *in vivo* from our previous studies 24. Prolonged exposure to the combination of FB23 and ibrutinib exhibits a synergistic effect, significantly enhancing the inhibition of cell proliferation in the MDA-MB-231^LMF3^ (Fig. 4A) and BT-549^LMF3^ (Fig. 4B) cell lines. Further, this combined treatment led to a significant reduction in the number of colonies formed compared to control or individual drug treatments in both cell lines (Fig. 4C and D). Regarding cell apoptosis, the combined use of FB23 and ibrutinib after 48 h of treatment increased the protein expression levels of caspase 3, cleaved caspase 3, and p53, and reduced protein expression levels of Bcl2 in MDA-MB-231^LMF3^ (Fig. 4E) and BT-549^LMF3^ (Fig. 4F) cells compared to control or single-drug treatments. These findings suggest that the combined treatment regimen may enhance apoptotic effects in these cells.

Our investigation encompassed cell migration and invasion. In migration assays, the combination of FB23 and ibrutinib significantly inhibited migration compared to FB23 alone, ibrutinib alone, or control in MDA-MB-231^LMF3^ cells after 24 h of incubation (Fig. 4G). In BT-549^LMF3^ cells, the combination similarly reduced migration relative to the single-agent treatments or control after 24 h (Fig. S3C). In invasion assays, the combination of FB23 and ibrutinib markedly decreased invasion compared to either FB23, ibrutinib, or the control in MDA-MB-231^LMF3^ cells (Fig. 4H). A comparable reduction in invasion was observed in BT-549^LMF3^ cells with the combination treatment relative to either FB23, ibrutinib, or the control (Fig. 4I). These results showed that the combination of FB23 and ibrutinib effectively suppresses the malignancy of lung metastasis of breast cancer.

### Inhibition of FTO and ibrutinib synergistically suppress *in vivo* growth and metastasis of breast cancer cells

After identifying that the combination of an FTO inhibitor and ibrutinib can function as an agent against breast cancer cells, we next used BALB/c-nu mice to investigate the specific effects of this combination on breast cancer progression *in vivo*. In these experiments, we used the shFTO cell line to inhibit FTO due to a lack of pharmacokinetic study of FB23. shFTO#2 was selected for FTO KD in MDA-MB-231 (Fig. 1G and S1B) and MDA-MB-231^LMF3^ (Fig. S4A and B) cells due to the greater efficiency.

**Figure 5:**
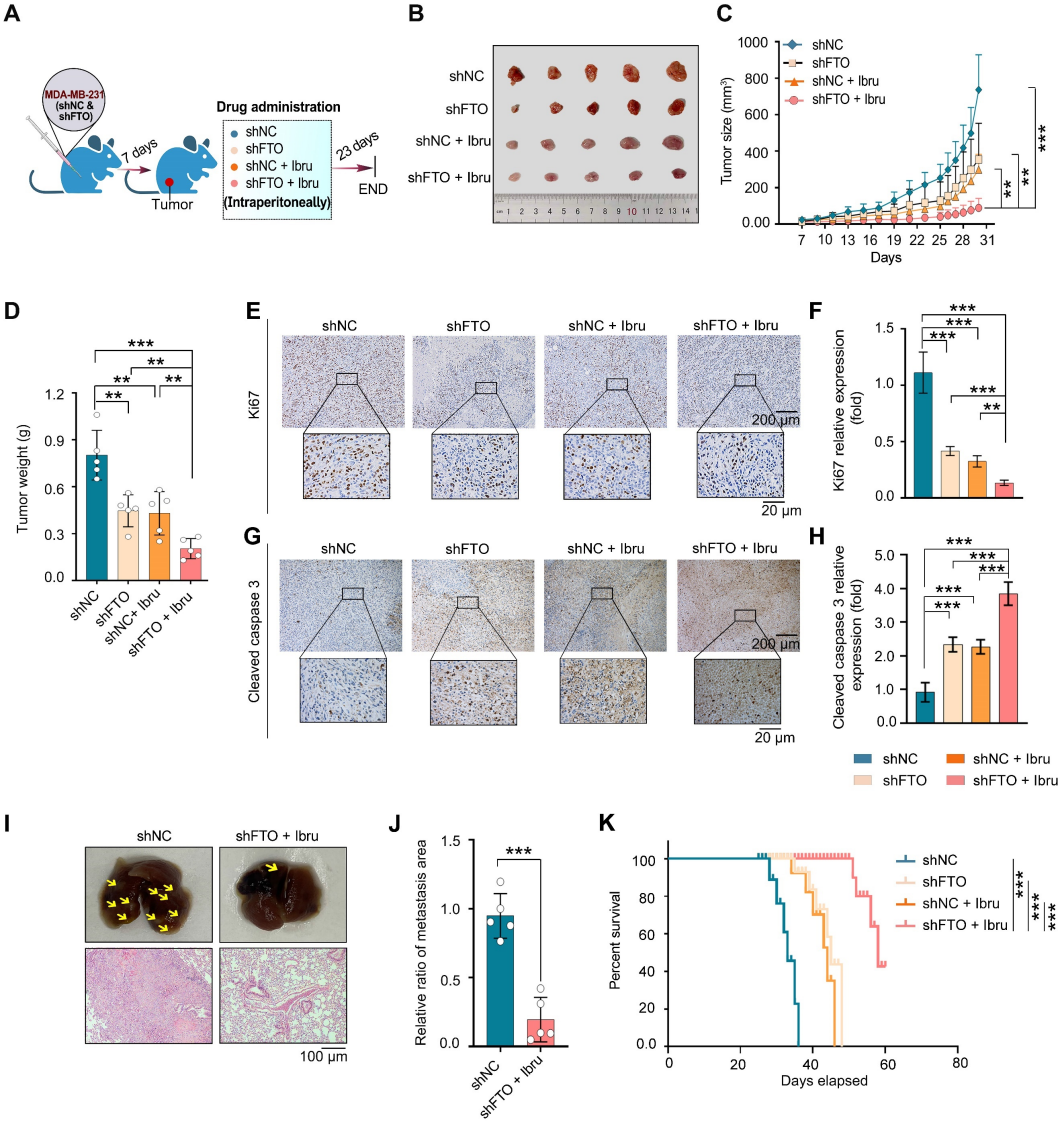
** Inhibition of FTO and ibrutinib synergistically suppresses tumor growth and metastasis *in vivo*. (A-D)** Evaluation of anticancer efficacy using a xenograft tumor model of MDA-MB-231 cells with inhibition of FTO and ibrutinib combination. (A) Schematic representation of the xenograft tumor model: MDA-MB-231 cells inoculated subcutaneously in mice, with treatment beginning on day 7. Mice were divided into four groups: shNC, FTO KD (shFTO), shNC plus ibrutinib, and shFTO plus ibrutinib. Daily intraperitoneal injections are administered for 23 days. (B) Image of a tumor dissected from nude mice. (C) Tumor growth curve, illustrating significant suppression of tumor growth in the combination therapy group. (D) Tumor weights measured 30 days post-transplant. *P* values were determined using one-way ANOVA followed by Tukey's multiple comparisons test (**P < 0.01, ***P < 0.001), with 5 mice per group. **(E-H)** Tumor-bearing mice were treated with shNC, shFTO, shNC plus ibrutinib, or shFTO plus ibrutinib. Tumor tissues were harvested on day 23 post-treatment. Representative IHC images and quantification of Ki67 (E and F) and cleaved caspase 3 (G and H) in tumor sections are shown. Statistical significance was assessed using one-way ANOVA followed by Tukey's multiple comparisons test (**P < 0.01, ***P < 0.001) (n = 5 mice per group). Scale bars = 20 μm and 200 μm. **(I**-**J)** Lung metastasis was assessed in a tail vein model using MDA-MB-231^LMF3^ cells (shNC or shFTO) injected into nude mice, with mice assigned to shNC and shFTO plus ibrutinib groups and receiving intraperitoneal injections from two weeks post-inoculation for four weeks. (I) Representative images of metastatic lung tumors and H&E staining of lung tissue sections showing metastatic foci. (J) The relative ratio of the metastasis area (n = 5 mice per group). Significance was assessed with exact *P* values (***P < 0.001) using a t-test. **(K)** Survival curve analysis in a tail-vein metastasis model using MDA-MB-231^LMF3^ cells (shNC or shFTO). Mice were assigned to four groups: shNC, shFTO, shNC plus ibrutinib, and shFTO plus ibrutinib. Intraperitoneal injections began two weeks post-inoculation. Kaplan-Meier survival curves display survival rates over time, showing significantly prolonged survival in the combination of shFTO plus ibrutinib compared to non-target and single-agent groups (6 mice per group). Statistical significance was determined by log-rank and Gehan-Breslow-Wilcoxon tests, with significance indicated by ***P < 0.001 denote the significant difference relative to shFTO plus ibrutinib group. The X-axis represents time in weeks post-inoculation, and the Y-axis shows the percentage of surviving mice.

The xenograft model in female nude mice by MDA-MB-231 cells (shNC or shFTO) into subcutaneous and administered four regimens: shNC, shFTO, shNC plus ibrutinib, and shFTO plus ibrutinib (Fig. 5A). Notably, the results showed that the tumor size (Fig. 5B), volume (Fig. 5C), and weight (Fig. 5D) of xenografts treated with shFTO and ibrutinib combination were significantly lower than those in the control group or when treated with either agent alone. No obvious weight loss was observed in mice (Fig. S4C). Immunohistochemical (IHC) confirmed a discernible decrease in the expression of the oncogenic protein FTO (Fig. S4D). Further, combined treatment with shFTO and ibrutinib resulted in a marked reduction in Ki67 expression, a well-established marker of cell proliferation, which was significantly greater than that observed with either treatment alone (Fig. 5E and F). Additionally, this combination markedly increased cleaved caspase 3 levels, a canonical marker of apoptosis, compared with single-agent treatments (Fig. 5G and H), consistent with *in vitro* findings.

A lung colonization model was developed by injecting MDA-MB-231^LMF3^ cells into the lateral tail vein to assess the effect of combined shFTO and ibrutinib treatment. The results demonstrated that treatment of shFTO with ibrutinib significantly reduced the number and size of lung tumors derived from MDA-MB-231^LMF3^ cells (Fig. 5I and J). Finally, we evaluated survival outcomes in a mouse model of lung metastasis following combination therapy (Fig. S4E). Mice receiving the combined treatment showed significantly prolonged survival compared to the control group and those treated with either agent alone (Fig. 5K). No significant weight change was noted in the mice under combination therapy (Fig. S4F). Collectively, animal studies indicate that combining FTO inhibition with ibrutinib synergistically reduces tumor growth and metastasis and improves survival, highlighting their potential in cancer treatment.

### Combination of FB23 and ibrutinib suppresses the c-Myc and E2F1 pathways in breast cancer cells

**Figure 6:**
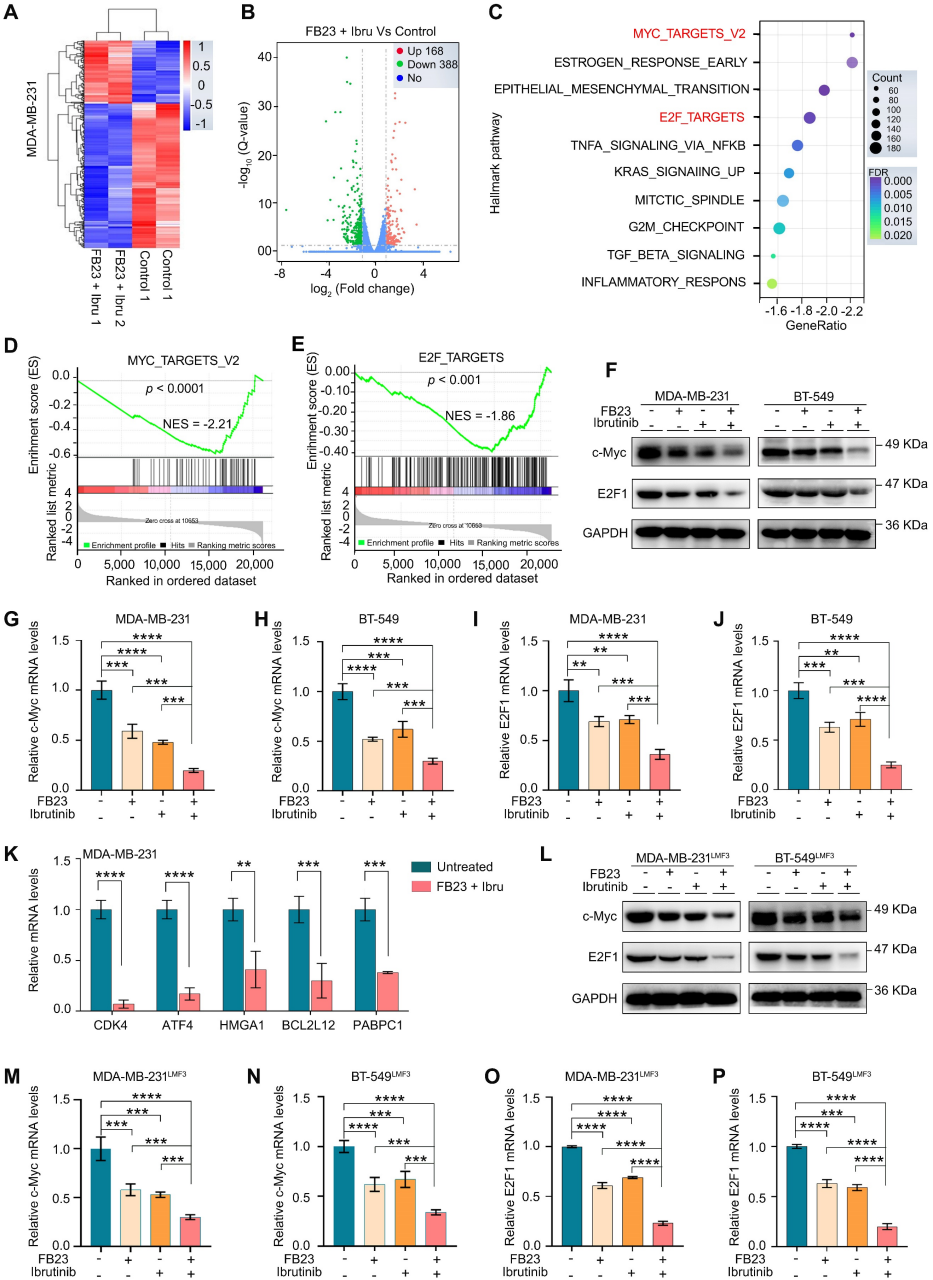
** Combination of FB23 and ibrutinib suppresses the c-Myc and E2F1 pathways. (A)** The heatmap provides an overview of the differentially expressed genes following treatment with combination drugs, offering a visual representation of gene expression changes. **(B)** Volcano plot depicting significantly upregulated and downregulated genes in drug combination-treated MDA-MB-231 cells. **(C)** Scattergram showing pathways downregulated by FB23 and ibrutinib combination, based on Gene Set Enrichment Analysis (GSEA). **(D**-**E)** GSEA highlighting significant negative enrichment of genes altered by the combination treatment of FB23 and ibrutinib in pathways associated with c-Myc (D) and E2F1 (E). **(F)** Protein expression levels of c-Myc and E2F1 in MDA-MB-231 and BT-549 cell lines following treatment with FB23, ibrutinib, and their combination. **(G**-**H)** The mRNA expression levels of c-Myc in MDA-MB-231(G) and BT-549 (H) cell lines following treatment with FB23, ibrutinib, and their combination. **(I**-**J)** The mRNA expression levels of E2F1 in MDA-MB-231(I) and BT-549 (J) cell lines following treatment with FB23, ibrutinib, and their combination. **(K)** The mRNA expression levels of downstream c-Myc target genes (CDK4, ATF4, HMGA1, BCL2L12, PABPC1) in MDA-MB-231 cells treated with a control and a combination of FB23 and ibrutinib. **(L)** Protein expression levels of c-Myc and E2F1 in MDA-MB-231^LMF3^ and BT-549^LMF3^ cell lines after treatment with FB23, ibrutinib, and their combination. **(M**-**N)** The mRNA expression levels of c-Myc in MDA-MB-231^LMF3^ (M) and BT-549^LMF3^ (N) cells following treatment with FB23, ibrutinib, and their combination. **(O**-**P)** The mRNA expression levels of E2F1 in MDA-MB-231^LMF3^ (O) and BT-549^LMF3^ (P) cells following treatment with FB23, ibrutinib, and their combination. Statistical significance for panels G-J and M-P is denoted by adjusted *P* values (**P < 0.01; ***P < 0.001; ****P < 0.0001), determined using one-way ANOVA followed by Tukey's multiple comparisons test.

To investigate the synergistic mechanism of FB23 and ibrutinib, we used RNA-seq to conduct a differential gene analysis between untreated and drug combination-treated groups. The heatmap of our analysis showed minimal differences between parallel control and combination-treated samples, reinforcing the reliability of our results (Fig. 6A). The volcano plot illustrated that 168 genes were upregulated and 388 genes were downregulated (Fig. 6B). Gene ontology (GO) enrichment analysis of these genes unveiled several enriched pathways such as receptor-ligand activity, receptor regulator activity, and extracellular matrix (Fig. S5A). Gene Set Enrichment Analysis (GSEA) indicated that the gene expression profile resulting from the combined treatment with FB23 and ibrutinib modulates several critical pathways involved in cell proliferation, migration, and invasion. Notably, this was evidenced by a significant downregulation of pathways, including hallmarks of MYC targets V2, estrogen response early, epithelial-mesenchymal transition, and E2F targets as seen with the combined FB23 and ibrutinib treatment (Fig. 6C). Among these pathways, the gene sets associated with the targets of c-Myc (Fig. 6D) and E2F (Fig. 6E) showed the most variation.

We then investigated the roles of c-Myc and E2F1 in the synergistic effect of FB23 and ibrutinib on the progression of breast cancer. Our results revealed that the combination significantly decreased the protein expression levels of c-Myc and E2F1 (Fig. 6F), as well as the mRNA expression levels of c-Myc in the MDA-MB-231 and BT-549 cell lines (Fig. 6G and H, respectively). Similarly, the mRNA expression levels of E2F1 were reduced in both MDA-MB-231 and BT-549 cell lines (Fig. 6I and J, respectively). Furthermore, the combination drug decreased the mRNA expression levels of downstream c-Myc targets, including CDK4, PABPC1, ATF4, BCL2L12, and HMGA 31, in MDA-MB-231 (Fig. 6K) and BT-549 (Fig. S5B) cells, which are important effectors of c-Myc inhibition-induced tumor suppression.

In the lung metastasis model of breast cancer, the combination of FB23 and ibrutinib led to a significant reduction in the protein expression levels of c-Myc and E2F1 (Fig. 6L), as well as the mRNA expression levels of c-Myc (Fig. 6M and N) in MDA-MB-231^LMF3^ and BT-549^LMF3^ cells. Likewise, the mRNA expression levels of E2F1 (Fig. 6O and P) decreased in MDA-MB-231^LMF3^ and BT-549^LMF3^ cells. This finding further supports that the combination of FB23 and ibrutinib effectively can suppress the expression of c-Myc and E2F1 and inhibit the related pathway in breast cancer cells.

### Downregulation of c-Myc and E2F1 is involved in the combination of FB23 and ibrutinib-suppressed malignancy of breast cancer cells

To investigate whether c-Myc and E2F1 downregulation was involved in combination of FB23 and ibrutinib-suppressed breast cancer progression, we explored the effects of c-Myc and E2F1 overexpression (OE), both individually and in co-overexpression. Overexpression of c-Myc and E2F1 was confirmed at the protein and mRNA levels (Fig. S6A-D).

**Figure 7:**
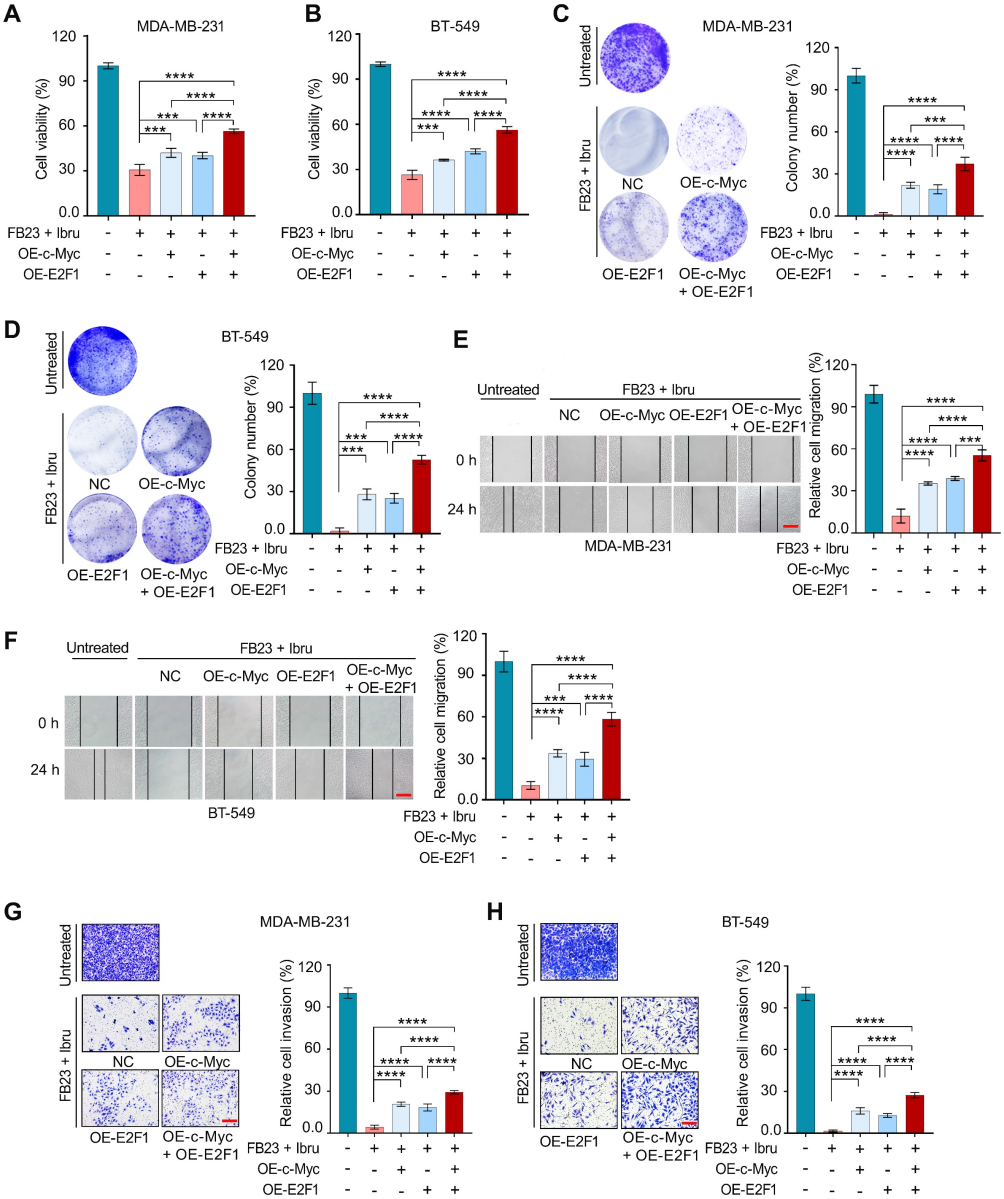
** Downregulation of c-Myc and E2F1 is involved in the combination of FB23 and ibrutinib-suppressed malignancy of breast cancer cells. (A**-**B)** CCK-8 assays of MDA-MB-231 (A) and BT-549 (B) cells transfected to overexpress c-Myc and E2F1 alone, or co-overexpress c-Myc/E2F1, then treated with a combination of FB23 and ibrutinib for 48 h. Groups included non-overexpressing (NC) and untreated controls. **(C**-**D)** Colony formation assays of MDA-MB-231 (C) and BT-549 (D) cells transfected to overexpress c-Myc and E2F1, either alone or co-overexpress c-Myc/E2F1, then treated with a combination of FB23 and ibrutinib for 12 days. Groups included non-overexpressing (NC) and untreated controls. **(E**-**F)** Migration assays of MDA-MB-231 (E) and BT-549 (F) cells transfected to overexpress c-Myc and E2F1, either singly or in co-overexpress c-Myc/E2F1, then treated with a combination of FB23 and ibrutinib 24 h. Groups included non-overexpressing (NC) and untreated controls. **(G**-**H)** Transwell invasion assays of MDA-MB-231 (G) and BT-549 (H) cells transfected to singly or co-overexpress c-Myc and E2F1, treated with combination of FB23 and ibrutinib for 48 h. Groups included non-overexpressing (NC) and untreated controls. Statistical significance is denoted by adjusted *P* values (**P < 0.01; ***P < 0.001; ****P < 0.0001), determined using one-way ANOVA followed by Tukey's multiple comparisons test. Scale bar = 100 µm.

First, overexpression of c-Myc and E2F1 individually increased cell proliferation; moreover, their co-overexpression led to an even greater increase in cell proliferation in both MDA-MB-231 (Fig. 7A) and BT-549 cells (Fig. 7B) after a 48 h incubation period. Colony formation assays revealed a significant increase in colony formation upon overexpression of c-Myc and E2F1, co-overexpression resulted in a much greater increase in colonies compared to individual overexpression in MDA-MB-231 (Fig. 7C) and BT-549 cells (Fig. 7D), suggesting the stronger influence on cell viability.

Consistently, overexpressing c-Myc and E2F1 significantly enhances the migratory capabilities of both MDA-MB-231 (Fig. 7E) and BT-549 cells (Fig. 7F). This enhancement is particularly pronounced when c-Myc and E2F1 are co-expressed. Invasion assays for both MDA-MB-231 (Fig. 7G) and BT-549 cells (Fig. 7H) demonstrate an increased invasive capacity, with co-overexpression leading to a significantly greater effect compared to individual overexpression. Thus, our data suggest that the downregulation of c-Myc and E2F1 contributes to the suppression of malignancy in breast cancer cells by the combination of FB23 and ibrutinib.

### Combination of FB23 and ibrutinib suppresses the expression of c-Myc and E2F1 via YTHDF2-induced decay of mRNA

**Figure 8:**
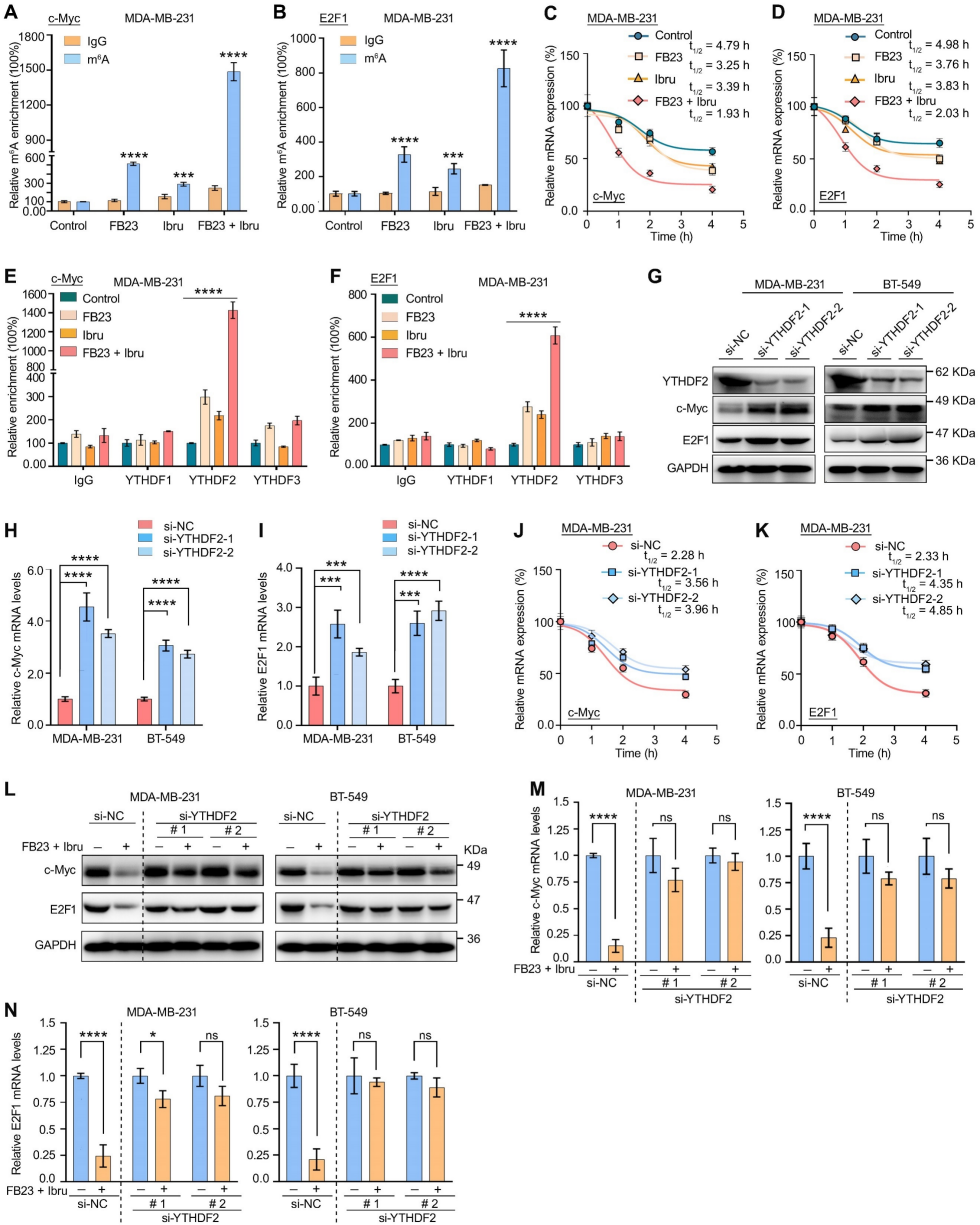
** Combination of FB23 and ibrutinib suppresses the expression of c-Myc and E2F1 via YTHDF2-induced decay of mRNA. (A**-**B)** The relative m^6^A enrichment of c-Myc (A) and E2F1 (B) mRNA in MDA-MB-231 cells treated with FB23, ibrutinib, or the combination of FB23 and ibrutinib, as well as the control group, for 24 h. m^6^A levels were measured using m^6^A-RIP-qPCR. **(C**-**D)** The stability of c-Myc (C) and E2F1 (D) mRNA in MDA-MB-231 cells was assessed following 24 h treatment with FB23, ibrutinib, or their combination, as well as a control group. After treatment, cells were incubated with Actinomycin D (Act-D) for 0-4 h to evaluate mRNA stability. **(E)** The relative enrichment of c-Myc in YTHDF1, YTHDF2, and YTHDF3 was assessed in MDA-MB-231 cells pre-treated with FB23, ibrutinib, or their combination, as well as the control group, for 24 h. Enrichment was analyzed using RIP-qPCR. **(F)** The relative enrichment of E2F1 in YTHDF1, YTHDF2, and YTHDF3 was assessed in MDA-MB-231 cells pre-treated with FB23, ibrutinib, or their combination, as well as the control group, for 24 h, was assessed by RIP-qPCR analysis. **(G)** Protein expression levels of c-Myc and E2F1 following YTHDF2 KD in cells transfected with si-YTHDF2-1/2 or si-NC for 24 h, then treated with a combination of FB23 and ibrutinib. **(H**-**I)** The mRNA expression levels of c-Myc (H) and E2F1 (I) following YTHDF2 KD in cells transfected with si-YTHDF2-1/2 or si-NC for 24 h, then treated with a combination of FB23 and ibrutinib. **(J**-**K)** Effect of YTHDF2 KD on the mRNA stability of c-Myc (J) and E2F1 (K) in MDA-MB-231 cells treated with a combination of FB23 and ibrutinib for 24 h, followed by incubation with Act-D for 0-4 h. **(L)** Protein expression levels of c-Myc and E2F1 in cells transfected with si-NC or si-YTHDF2-1/2 for 12 h, followed by treatment with or without a combination of FB23 and ibrutinib. **(M)** c-Myc mRNA expression levels in cells transfected with si-NC or si-YTHDF2-1/2 for 12 h, then treated with or without a combination of FB23 and ibrutinib for 24 h. **(N)** E2F1 mRNA expression levels in cells transfected with si-NC or si-YTHDF2-1/2 for 12 h, followed by treatment with or without a combination of FB23 and ibrutinib for 24 h. The indicated *P* values (*P < 0.05, ***P < 0.001, ****P < 0.0001) in panels A, B, M, and N were determined using Student's t-test. For panels E, F, H, and I, *P* values (***P < 0.001, ****P < 0.0001) were determined using one-way ANOVA followed by Dunnett's post hoc multiple comparisons test.

The mechanisms responsible for the regulation of c-Myc and E2F1 expression through the combination of FB23 and ibrutinib were further studied. We have performed m^6^A-RIP-qPCR analysis for c-Myc and E2F1 in cells treated with FB23, ibrutinib, and the combination of FB23 and ibrutinib. The results have confirmed a more significant increase in m⁶A enrichment of c-Myc and E2F1 mRNAs upon combination treatment, indicating that co-treatment with FB23 and ibrutinib has enhanced m⁶A modification of c-Myc (Fig. 8A and S7A) and E2F1 (Fig. 8B and S7B) mRNAs in MDA-MB-231 and BT-549 cells. Previous studies indicated that m^6^A may negatively regulate the mRNA stability of c-Myc and E2F1 in cancer cells 9, 32. Our data showed that the combination of FB23 and ibrutinib in MDA-MB-231 and BT-549 cells markedly decreased the stability of c-Myc (Fig. 8C and S7C) and E2F1 (Fig. 8D and S7D) mRNA compared with FB23, ibrutinib, or control treatment. These findings suggest that the downregulation of c-Myc and E2F1 in breast cancer cells by combined FB23 and ibrutinib treatment is likely attributable to reduced transcript stability.

YTH domain family proteins—particularly YTHDF1, YTHDF2, and YTHDF3—have emerged as the most extensively studied m⁶A readers 33. Among them, YTHDF2 was one of the most important m^6^A reader proteins to induce the degradation of target mRNA 34. We then executed a RIP assay using an antibody that targets the m^6^A reader YTHDF1/2/3. The results revealed significant and specific binding of YTHDF2 to c-Myc and E2F1 transcripts, markedly stronger than that observed for YTHDF1 or YTHDF3. Notably, YTHDF2 binding to c-Myc (Fig. 8E and S7E) and E2F1 (Fig. 8F and S7F) was substantially enhanced in MDA-MB-231 and BT-549 cells treated with a combination of FB23 and ibrutinib. To confirm the critical role of YTHDF2's m^6^A-binding function in regulating c-Myc and E2F1 protein expression, we silenced YTHDF2 using siRNAs (si-YTHDF2-1 and si-YTHDF2-2) (Fig. 8G and S7G). This silencing resulted in increased protein (Fig. 8G) and mRNA (Fig. 8H and I) levels of c-Myc and E2F1 in MDA-MB-231 and BT-549 cells treated with the combination of FB23 and ibrutinib. Further, the rates of stability of c-Myc (Fig. 8J and S7H) and E2F1 (Fig. 8K and S7I) mRNAs increased upon YTHDF2 depletion, indicating that YTHDF2 is essential for the stabilization of c-Myc and E2F1 transcripts. Moreover, depleting YTHDF2 can mitigate the effects of combining FB23 and ibrutinib, resulting in increased protein (Fig. 8L) and mRNA (Fig. 8M and N) levels of c-Myc and E2F1 in MDA-MB-231 and BT-549 cells. Together, our findings indicate that m^6^A changes resulting from the combination of FB23 and ibrutinib suppressed the expression of c-Myc and E2F1 via YTHDF2-induced decay of mRNA.

### The clinical potential of FTO-BTK/c-Myc-E2F1 pathways on breast cancer

**Figure 9:**
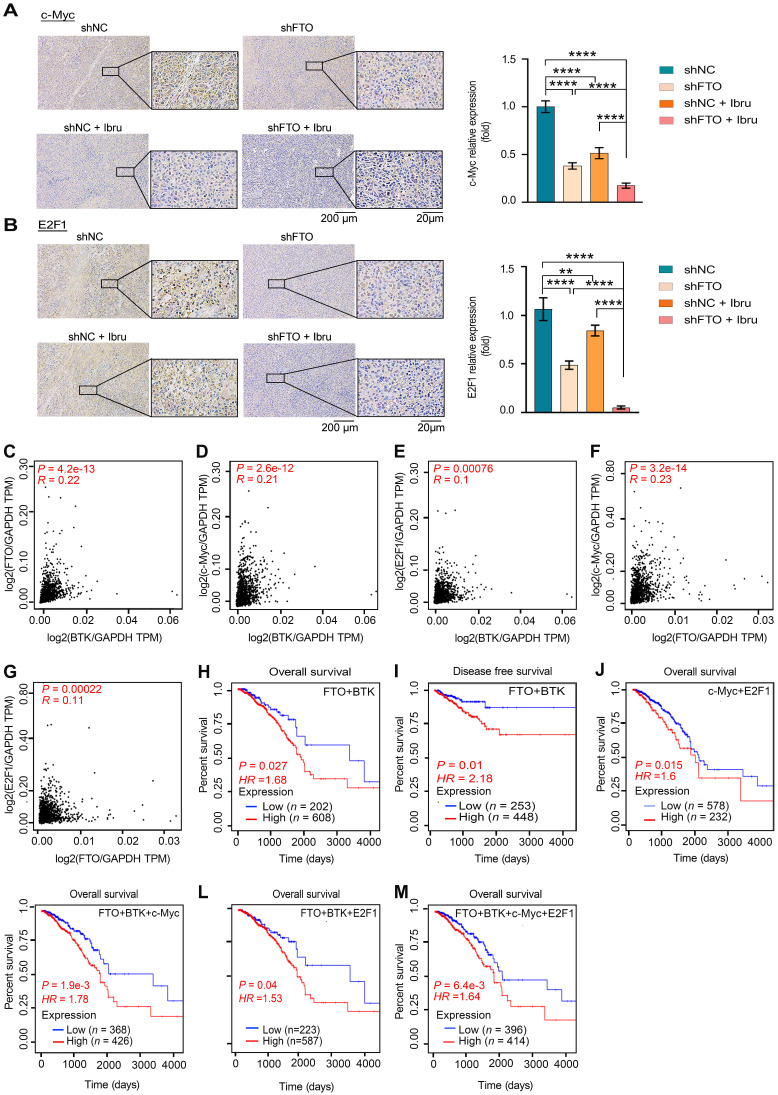
** The clinical potential of FTO-BTK/c-Myc-E2F1 pathways on breast cancer. (A**-**B)** IHC staining highlights c-Myc (A) and E2F1 (B) expression in tumor tissues of tumor-bearing mice. *P* values (**P < 0.01; ****P < 0.0001) were calculated using one-way ANOVA followed by Tukey's multiple comparisons post hoc test. Scale bars represent 20 and 200 μm. **(C**-**G)** Scatterplots showing pairwise correlations of relative mRNA expression between BTK and FTO (C), BTK and c-Myc (D), BTK and E2F1 (E), FTO and c-Myc (F), and FTO and E2F1 (G) in breast cancer tissues from GEPIA 2.0 database. **(H)** The Kaplan-Meier survival curves of OS based on FTO and BTK expression in breast cancer patients from TCGA database. **(I)** The Kaplan-Meier survival curves of DFS based on FTO and BTK expression in breast cancer patients from TCGA database. **(J)** The Kaplan-Meier survival curves of OS based on c-Myc and E2F1 expression in breast cancer patients from TCGA database. **(K)** The Kaplan-Meier survival curves of OS are based on FTO, BTK and c-Myc expression in breast cancer patients from TCGA database. **(L)** The Kaplan-Meier survival curves of OS based on FTO, BTK and E2F1 expression in breast cancer patients from TCGA database. **(M)** The Kaplan-Meier survival curves of OS based on FTO, BTK, c-Myc and E2F1 expression in breast cancer patients from TCGA database.

Firstly, we assessed the *in vivo* effects of inhibition of FTO-BTK on c-Myc and E2F1 expression levels in xenograft models. IHC revealed a marked reduction in the protein levels of the oncogenes c-Myc (Fig. 9A) and E2F1 (Fig. 9B) when treated with a combination of shFTO and ibrutinib. To further validate the clinical relevance of the strong correlation between FTO and BTK in breast cancer, and to investigate the potential impact of FTO-BTK modulation on c-Myc and E2F1 expressions, we utilized the GEPIA 2.0 database for correlation analysis between FTO/BTK expression levels and the expression of c-Myc and E2F1, revealing significant positive correlations (Fig. 9C-G). This underscores the potential clinical importance of targeting the FTO-BTK axis in modulating oncogenic expressions. To assess the impact of the FTO-BTK/c-Myc-E2F1 axis expression levels on the survival of breast cancer patients, we using SangerBox.2 for multifactorial survival analysis, we discovered that lower expression levels of the FTO and BTK axis were significantly associated with improved OS (Fig. 9H) and disease-free survival (DFS) (Fig. 9I) in breast cancer patients. This highlights the potential benefits of targeting both FTO and BTK in combination therapies. Further multifactorial survival analysis showed that low expression levels of the c-Myc and E2F1 axis significantly improved OS in breast cancer patients (Fig. 9J). In line with this, we investigated whether FTO and BTK influence OS in breast cancer patients through c-Myc and E2F1. Analysis revealed that lower expression levels of FTO-BTK/c-Myc were significantly associated with improved OS in breast cancer patients (Fig. 9K). Similarly, lower expression levels of FTO-BTK/E2F1 also significantly enhanced OS in breast cancer patients (Fig. 9L). Finally, multifactorial survival analysis revealed that lower expression levels of FTO-BTK/c-Myc-E2F1 significantly improved OS in breast cancer patients (Fig. 9M). In sum, FTO and BTK are important regulators of c-Myc and E2F1 accumulation in breast cancer, and their induction of low expression levels is a major factor in improving the survival outcomes of breast cancer patients.

## Discussions

**Figure 10:**
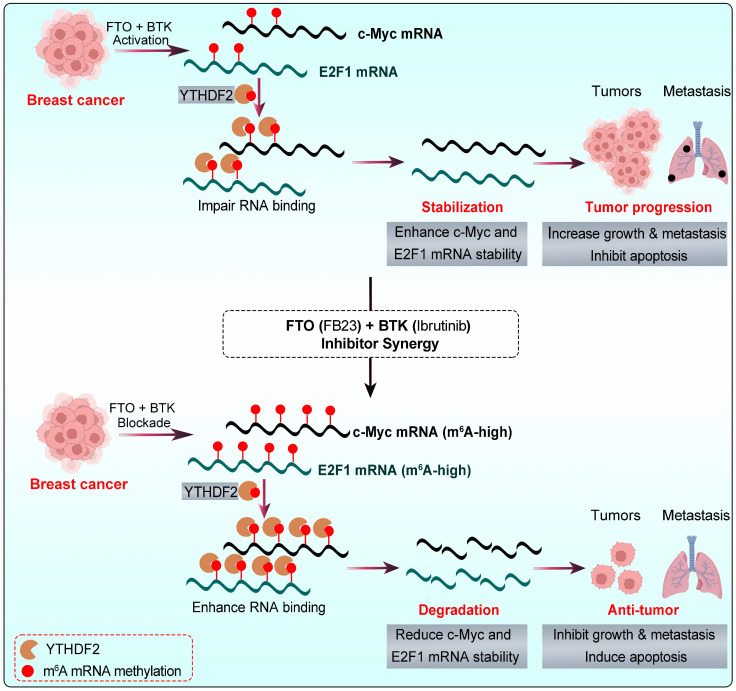
The graphic illustration of the mechanism by which the combination of FB23 (FTO inhibitor) and ibrutinib (BTK inhibitor) suppresses c-Myc and E2F1 expression via YTHDF2-mediated mRNA decay.

Targeted therapies, revolutionizing cancer care in the past decade, offer personalized options with impressive specificity. However, single-drug approaches face limitations like drug resistance and adherence issues 17. Therefore, multi-target drugs, which simultaneously inhibit two targets, are emerging as a potent and effective strategy in cancer therapy. This is crucial for breast cancer, a complex, aggressive disease with high recurrence and metastasis rates and worse outcomes 4. FTO dysregulation causes tumor growth by impacting m^6^A-dependent processes 9, 12, 13. Upregulated FTO contributes to cancer progression, including breast cancer, by increasing cell growth, promoting stem cell self-renewal, altering cancer immunity and metabolism by modifying target mRNA stability 9, 10, 13, 35, 36. Research has focused on developing small-molecule FTO inhibitors, such as R-2HG 26, FB23, and FB23-2 12, which have shown antitumor effects in AML and show promising potential for broader cancer treatment 10, 35, 36. However, the efficacy of some FTO inhibitors as standalone treatments may be suboptimal 9, 13, highlighting the need for combination therapies to enhance their antitumor activity. Herein, this study unveils the synergistic antitumor effect of FB23 and ibrutinib in breast cancer, both *in vitro* and *in vivo*. This synergy likely stems from inactivated signaling pathways and downregulation of c-Myc and E2F1 expression (Fig. 10).

Recent studies demonstrate that combining FTO inhibitors with other targeted therapies can potentially enhance their synergistic antitumor effects 9. For instance, the proven synergy between rhein, an FTO inhibitor, and nilotinib, a TKI, significantly improves therapeutic outcomes in AML 14. The FB23-everolimus combination synergistically inhibits pancreatic neuroendocrine tumors 15; similarly, the combination of FB23 and BX-912 targets FTO and the PDK1-AKT pathway to suppress breast cancer 16. In this study, we have demonstrated that while FB23 alone exhibits limited antitumor activity against breast cancer, its combination with ibrutinib presents a notably effective treatment strategy. This synergistic effect significantly curtails cell survival and proliferation over various treatment durations and crucially inhibits colony formation, underscoring the potential of this combination as a broad-spectrum antiproliferative agent. Notably, the combined treatment of FB23 and ibrutinib surpasses the efficacy of either drug alone in blocking key cellular processes such as migration and invasion, suggesting enhanced therapeutic utility against aggressive cancer phenotypes. Ibrutinib alone inhibits key cellular processes, including proliferation, colony formation, migration, and invasion 22. Similarly, FTO inhibitors have been shown to exert inhibitory effects on these processes in our data and previous studies 9, thereby underpinning the observed synergy. Reports have established that FTO inhibitors can trigger apoptosis in cancer cells 9, 13, 37. Similarly, ibrutinib has been shown to induce apoptosis 38, 39. Our results demonstrate that combination of FB23 and ibrutinib acts synergistically to enhance apoptosis, further, their combination effectively inhibits metastasis in LMF3 breast cells.

Mechanistically, c-Myc and E2F1 are potent oncogenes that are critical drivers of cancer progression, impacting growth, cell cycle, proliferation, and metastasis across various cancers 40, 41. c-Myc was reported to be highly expressed, which leads to the occurrence and development of breast cancer 42. High E2F1 expression in breast cancer tissues is linked to poor prognosis, promoting tumor cell viability, metastasis, and cell cycle progression 43-45. Our result shows that reducing c-Myc and E2F1, which are vital in m^6^A-regulated cancer progression, is crucial in the combination therapy of FB23 and ibrutinib. Overexpressing c-Myc and E2F1 reduces the effectiveness of FB23 and ibrutinib in treating cancer. m^6^A is a crucial regulator, playing key roles in tissue development, stem cell processes, biological rhythm, and DNA repair 8, 46, 47. The post-transcriptional regulator FTO influences carcinogenic networks, including breast cancer 9, 10. This study revealed that the combined use of FB23 and ibrutinib increases m^6^A accumulation on c-Myc and E2F1 transcripts, reducing c-Myc and E2F1 mRNA stability and signaling, thereby enhancing antitumor effects. These findings are partly consistent with previous reports linking FTO to the regulation of c-Myc and E2F1. For instance, FTO has been shown to increase cervical cancer cell migration and proliferation by facilitating the translation of the oncogenic transcripts E2F1 and c-Myc 32. Additionally, FTO inhibitors have been linked to reduced c-Myc mRNA stability 26. FTO is also recognized as a crucial gene that interferes with the m^6^A modification of E2F1 48, and overexpression of FTO has been found to decrease m^6^A levels in E2F1, highlighting its critical role in the progression of lung cancer 49.

YTHDF2 is a major m^6^A reader responsible for the decay of m^6^A-modified mRNA transcripts 34. It is known to bind c-Myc and E2F1 mRNA, playing a pivotal role in regulating c-Myc and E2F1 expression 26, 50. Our results revealed an increase in the m^6^A-binding ability of YTHDF2, and the KD of YTHDF2 noticeably increased the stability and expression of c-Myc and E2F1 transcripts in breast cancer cells. FB23 and ibrutinib cooperatively enhance m^6^A modification, destabilizing c-Myc and E2F1 transcripts through YTHDF2, thereby amplifying their antitumor effects. In our *in vivo* studies, the combination of FTO inhibitors with ibrutinib markedly reduced tumor growth and lung metastasis in breast cancer and improved survival. IHC confirmed decreased c-Myc and E2F1 protein levels, consistent with their destabilization. Supporting clinical data further revealed significant positive correlations between FTO/BTK expression levels and c-Myc/E2F1 expression. Collectively, these findings highlight dual inhibition of FTO and BTK as a promising therapeutic strategy for maintaining low c-Myc and E2F1 expression levels, thereby improving survival outcomes and guiding future clinical management of breast cancer.

To sum up, the combination of FB23, an FTO inhibitor, and ibrutinib, a BTK inhibitor, significantly and synergistically suppresses breast cancer tumorigenicity. This suppression is achieved through the downregulation of c-Myc and E2F1, which is mediated by increased m⁶A modification, a process that is presumably associated with YTHDF2 (Fig. 10). Given these findings, FTO is identified as a promising therapeutic target in breast cancer. Furthermore, the combination of FB23 and ibrutinib warrants further investigation as a prospective treatment strategy, and our preclinical data support its evaluation in clinical trials.

## Supplementary Material

Supplementary figures and tables, materials and methods.

## Abbreviations

AML: acute myeloid leukemia
BTK: Bruton's tyrosine kinase
CCK-8: cell counting kit‑8
CI: combination index
DFS: disease-free survival
ER: estrogen receptor
FBS: fetal bovine serum
FTO: fat mass and obesity-associated protein
GSEA: gene set enrichment analysis
GO: gene ontology
HER2: human epidermal growth factor receptor 2
IC_50_: half maximal inhibitory concentration
IHC: immunohistochemistry
KD: knockdown
LM: lung metastasis
LMBC: lung metastatic breast cancer
MA: meclofenamic acid
m^6^A: *N*^6^-methyladenosine
mRNA: messenger RNA
OE: overexpression
OS: overall survival
PI: propidium iodide
PMSF: phenylmethylsulfonyl fluoride
PR: progesterone receptor
RIPA: radioimmunoprecipitation assay
RIP-RT-qPCR: RNA immunoprecipitation real-time qPCR
RT-PCR: real-time polymerase chain reaction
TCGA: The Cancer Genome Atlas
TKIs: tyrosine kinase inhibitors
TNBC: triple-negative breast cancer
