# Supplementary material for: Combination of FTO and BTK inhibitors synergistically suppresses the malignancy of breast cancer cells
Source: Int J Biol Sci. 2025 Oct 27;21(15):7063–85. doi: 10.7150/ijbs.117999 (PMC12631244; doi:10.7150/ijbs.117999)
Supplement: Supplementary file 1 — Supplementary figures and tables, materials and methods. [file ijbsv21p7063s1.pdf]

Supplementary data for:

Combination of FTO and BTK inhibitors synergistically suppresses the malignancy of breast cancer cells

Abdulaziz et al.

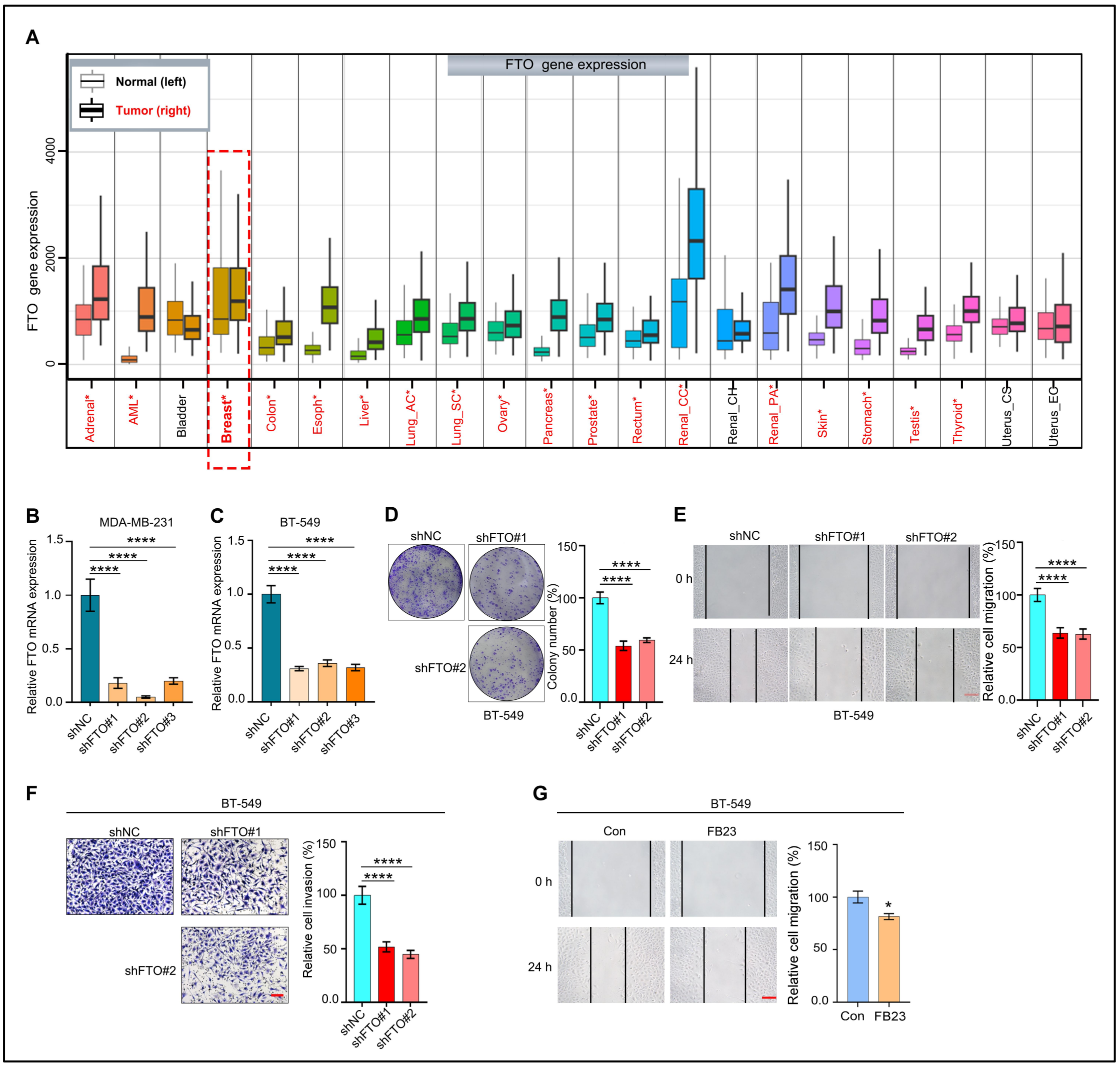

Supplementary Fig.1 FTO serves as a potential target for breast cancer therapy.

(A) The web tool TNMplot (https://tnmplot.com/analysis/) conducted a pan-cancer analysis that displays the mRNA expression levels of the FTO gene across various tissues in both normal and tumor samples. Areas with statistically significant differences determined by the Mann-Whitney U test are highlighted in red and marked with an asterisk**.**

(B-C) Validation of stable knockdown of FTO in MDA-MB-231(B) and BT-549 (C) cells by lentiviral shRNA sequences (shFTO#1, #2, #3) at mRNA levels.

(D) Colony formation assays assessing the impact of FTO KD on BT-549 cell proliferation for 12 days.

(E) Migration assays evaluating the effect of FTO KD on the migration of BT-549 cells.

(F) Invasion assays evaluating the effect of FTO KD on the invasion of BT-549 cells.

(G) Migration ability of BT-549 cells treated with FB23 for 24 h, compared to untreated control groups.

Statistical analysis was performed using t-tests or one-way ANOVA followed by Dunnett's test. Data are presented as mean ± SD, with exact P values (**$P < 0.01$; ****$P < 0.0001$) reported. Scale bar = 100 µm.

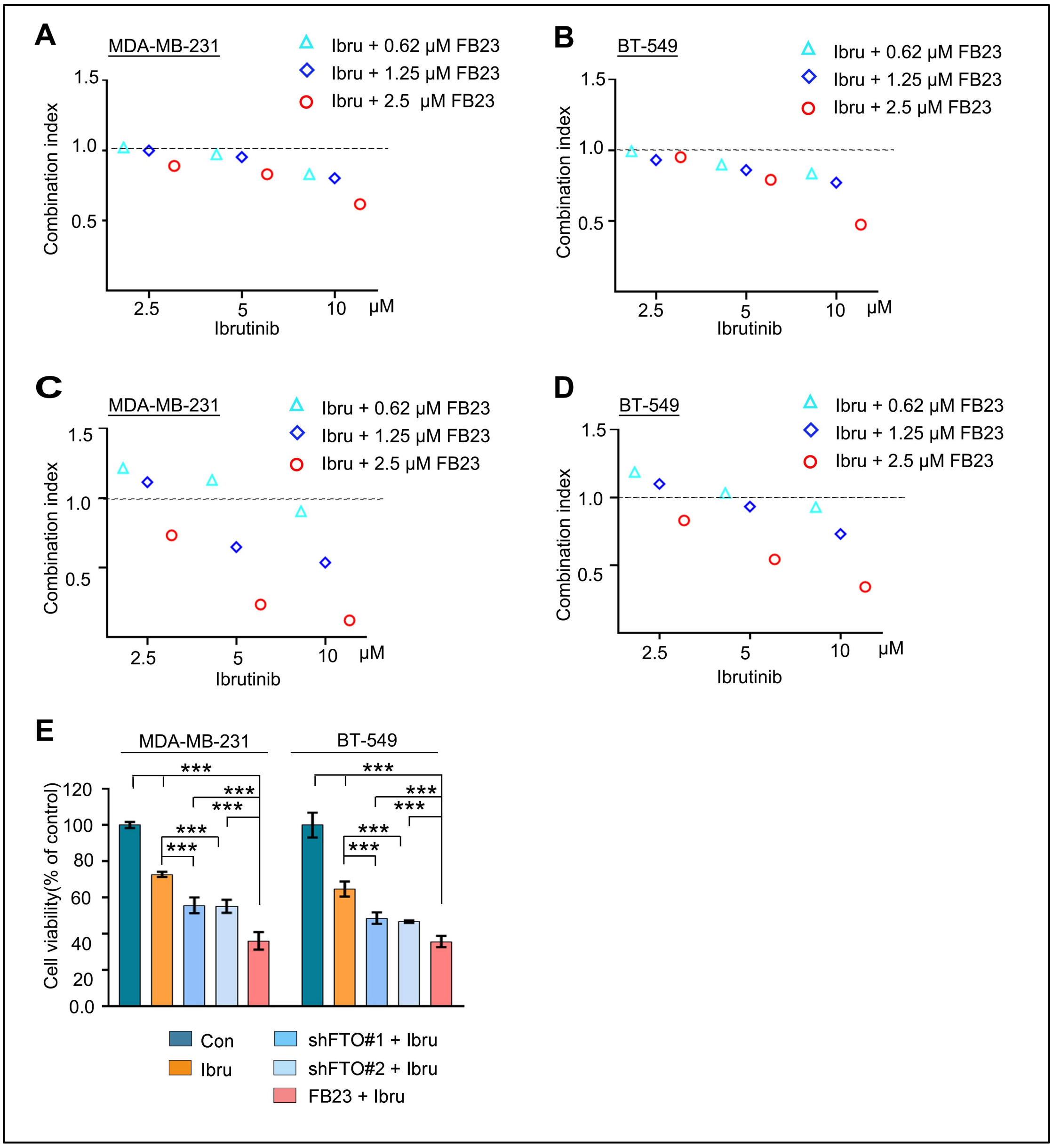

Supplementary Fig. 2. Dual inhibition of FTO and BTK synergistically targets breast cancer.

(A) CI analysis of the synergistic effect of FB23 and ibrutinib on proliferation in MDA-MB-231 cell lines (related to Fig. 2E).

(B) CI analysis of the synergistic effect of FB23 and ibrutinib on proliferation in BT-549 cell lines (related to Fig. 2F).

(C) CI analysis of the synergistic effect of FB23 and ibrutinib on colony formation assays to assess the proliferation in MDA-MB-231 cell lines for 12 days (related to Fig. 2G).

(D) CI analysis of the synergistic effect of FB23 and ibrutinib on colony formation assays to assess the proliferation in BT-549 cell lines for 12 days (related to Fig. 2H).

(E) Effects of FTO knockdown on the sensitivity of MDA-MB-231 and BT-549 cells to ibrutinib, as assessed by cell viability after 48 h. *P* values were determined by one-way ANOVA followed by Tukey's multiple comparisons test. ***$P < 0.001$

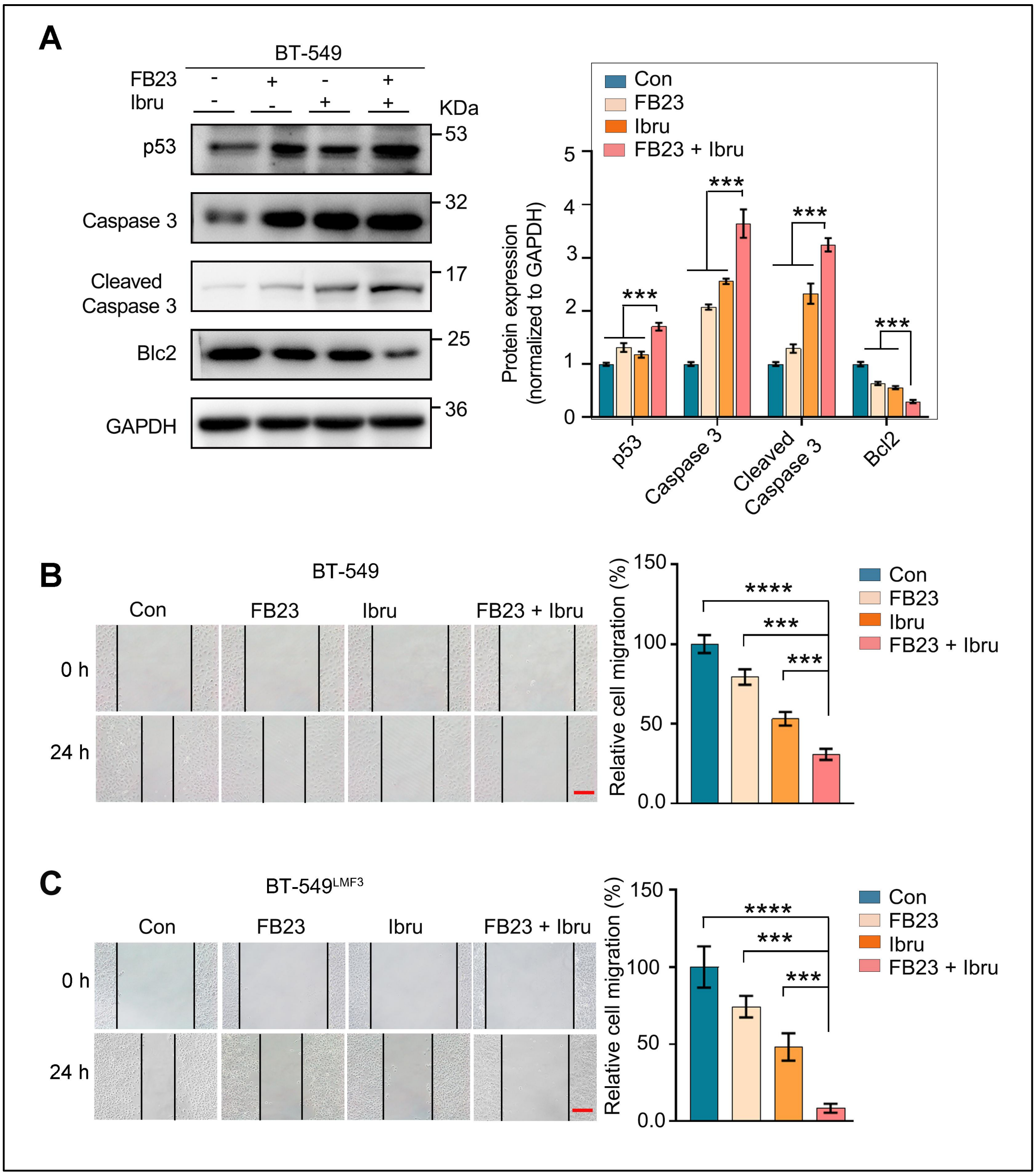

Supplementary Fig. 3. FB23 and ibrutinib synergistically inhibit the malignancy of breast cancer and LMBC cells.

(A) Protein expression levels of apoptosis markers—p53, caspase 3, cleaved caspase 3, and Bcl2—in BT-549 cells following 48 h of treatment with FB23 (2.5 μM), ibrutinib (10 μM), or their combination.

(B) The migration of BT-549 cells treated with ibrutinib alone, FB23 alone, or a combination of ibrutinib and FB23 for 24 h.

(C) The migration assay of BT-549^{LMF3} cells treated with ibrutinib alone, FB23 alone, or a combination of ibrutinib and FB23.

Statistical analysis was conducted using one-way ANOVA followed by Dunnett's multiple comparisons test. Data are presented as mean ± SD, with *P* values (***$P < 0.001$; ****$P < 0.0001$) provided. Scale bar = 100 μm.

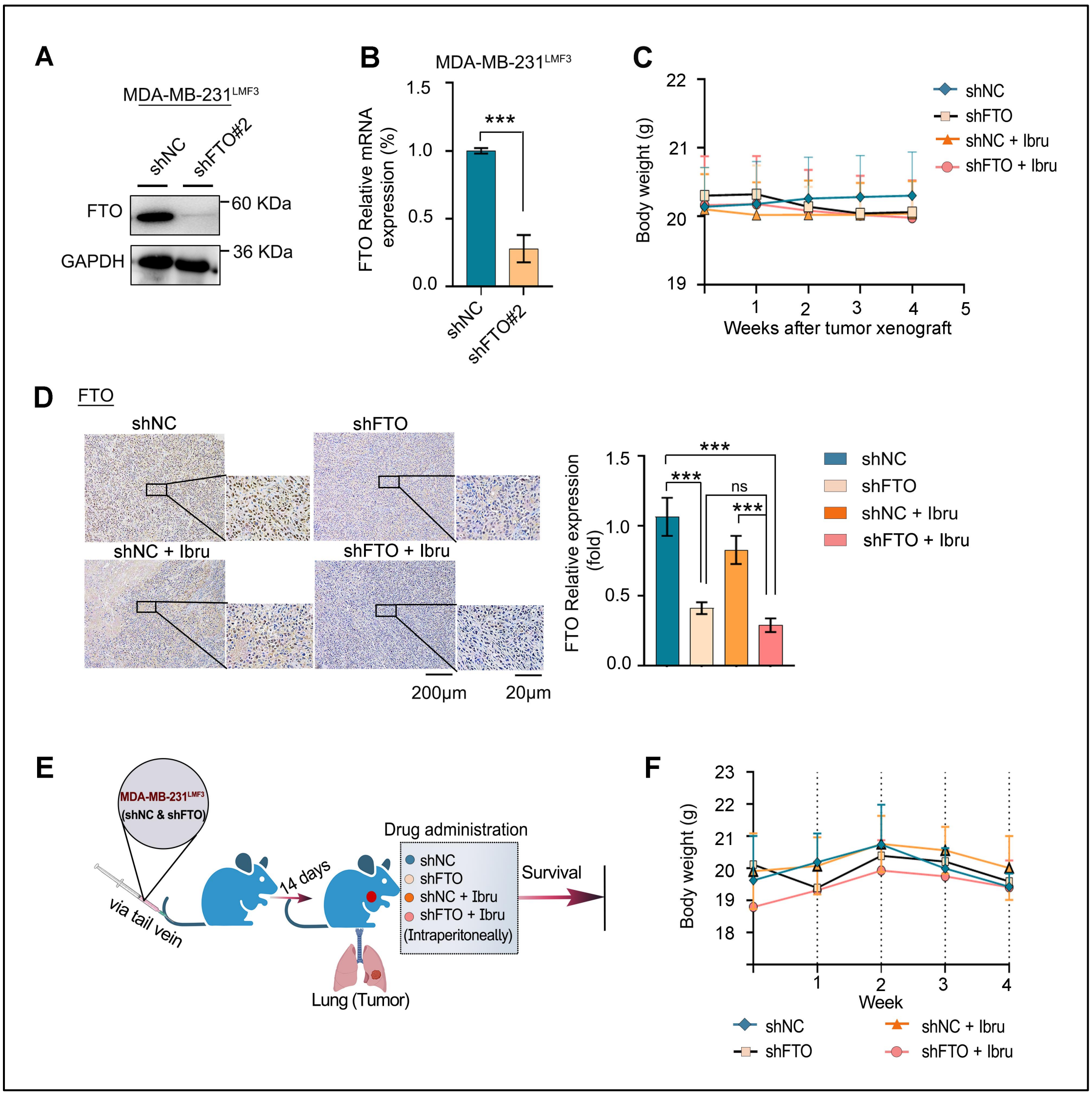

Supplementary Fig. 4. Inhibition of FTO and ibrutinib synergistically suppress tumor growth and metastasis *in vivo.*

(A-B) Confirmation of stable FTO knockdown in MDA-MB-231^{LMF3} cells using the lentiviral shRNA sequence (shFTO#2) at both protein and mRNA levels.

(C) Body weight measurements of the following groups in MDA-MB-231 xenograft models at specified intervals: shNC, shFTO, shNC + ibrutinib, and shFTO + ibrutinib.

(D) IHC staining demonstrating FTO expression in tumor tissues.

(E) Schematic illustration of the treatment strategy aimed at analyzing survival benefits. Cells, shNC or shFTO, were divided into four groups: shNC, shFTO, shNC + ibrutinib, and shFTO + ibrutinib. Intraperitoneal injections started two weeks post-inoculation.

(F) Body weight measurements in metastatic lung tumor models for the following groups: shNC, shFTO, shNC + ibrutinib, and shFTO + ibrutinib.

Statistical significance: Adjusted *P* values (***$P < 0.001$) were calculated using t-tests for (B) and one-way ANOVA followed by Tukey's multiple comparisons test for (D).

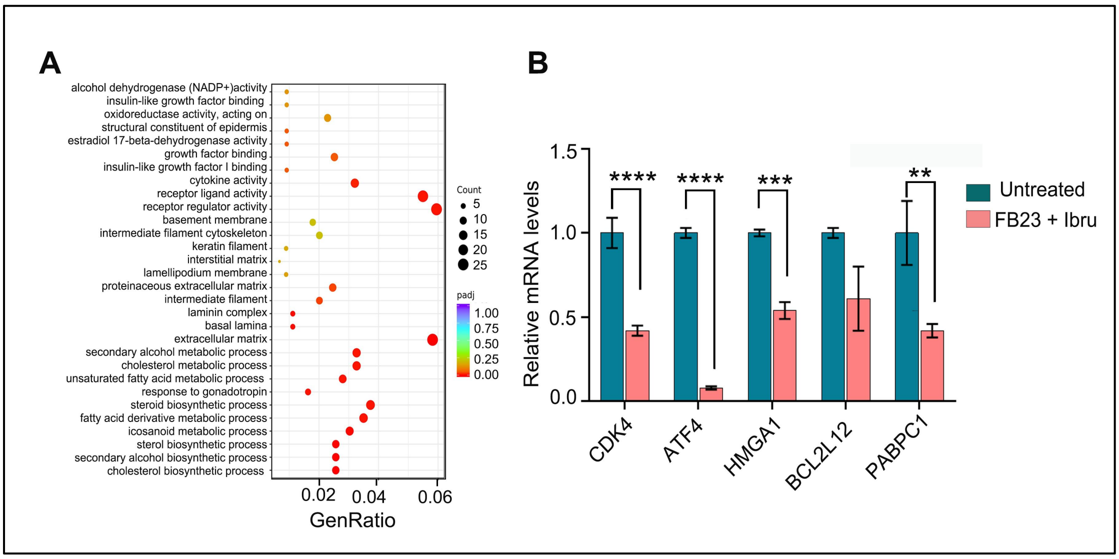

Supplementary Fig. 5. Combination of FB23 and ibrutinib suppresses the c-Myc and E2F1 pathways in breast cancer cells.

(A) GO enrichment analysis of biological processes in assembled unigenes from the transcriptome induced by the combination of FB23 and ibrutinib.

(B) The mRNA expression levels of downstream c-Myc target genes (CDK4, ATF4, HMGA1, BCL2L12, PABPC1) in BT-549 cells treated with either a control or the combination of FB23 and ibrutinib. The data, represented as mean ± SD, were analyzed using a t-test, with *P* values (**$P < 0.01$; ***$P < 0.001$; ****$P < 0.0001$) denoting significance.

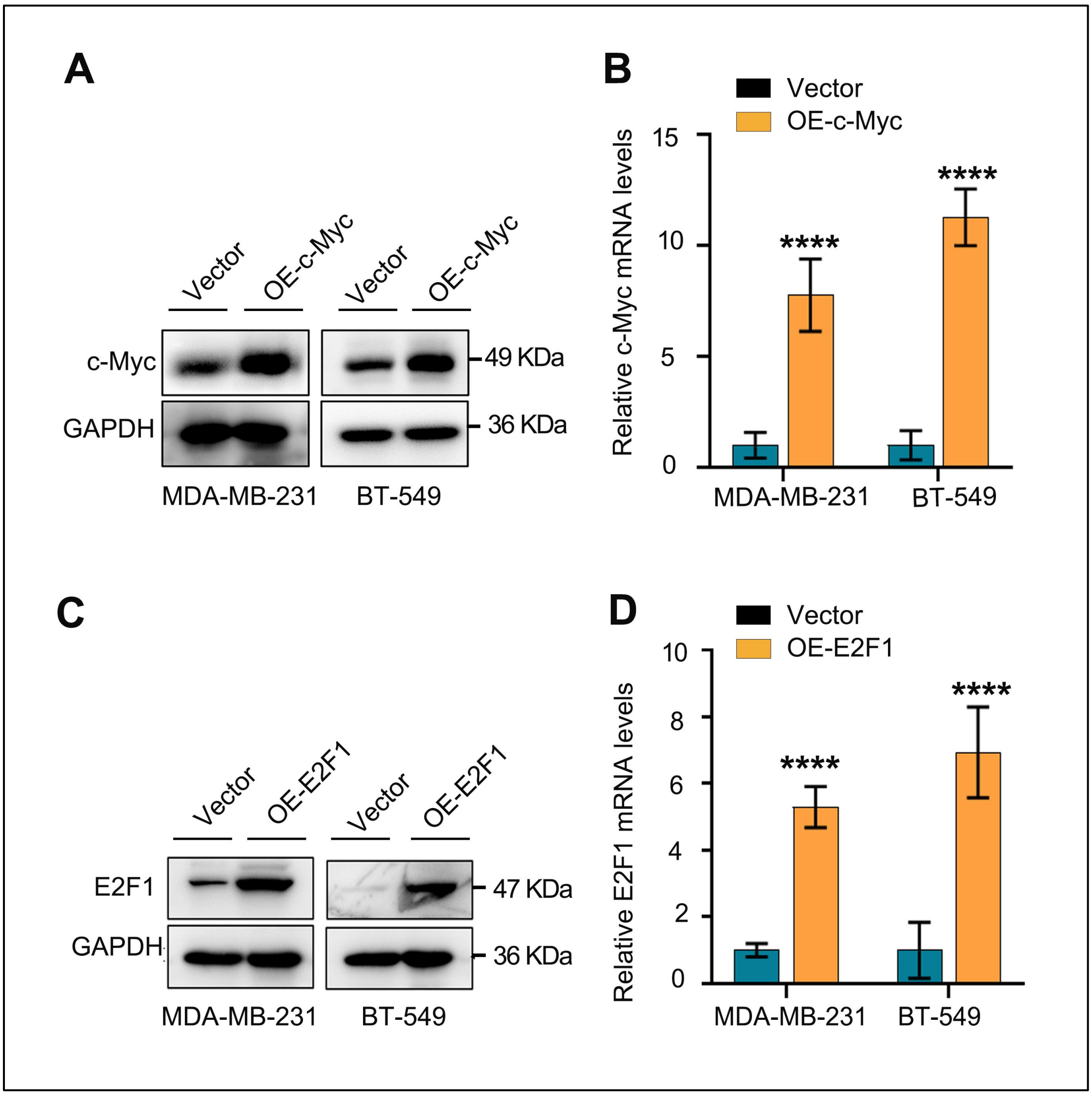

Supplementary Fig. 6. Downregulation of c-Myc and E2F1 is involved in combination of FB23 and ibrutinib-suppressed malignancy of breast cancer cells.

(A-B) Confirmation of c-Myc overexpression in MDA-MB-231 (A) and BT-549 (B) cells at both protein and mRNA levels, assessed using Western blotting and RT-qPCR, respectively.

(C-D) Confirmation of E2F1 overexpression in MDA-MB-231(C) and BT-549 (D) cells at both protein and mRNA levels, using Western blotting and RT-qPCR, respectively.

Data are presented as mean ± SD, with statistical significance denoted by ****P < 0.0001, calculated using a t-test.

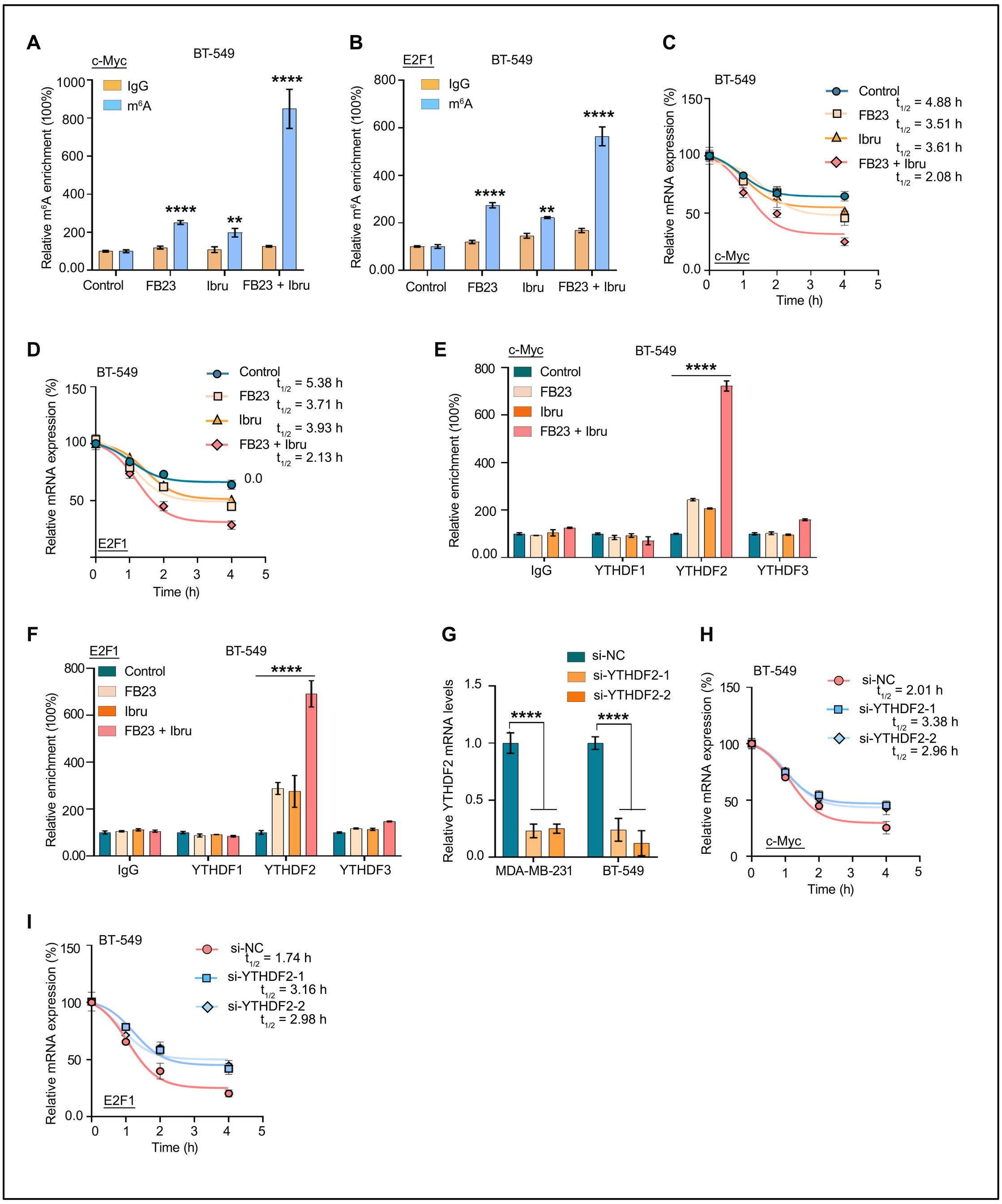

Supplementary Fig. 7. Combination of FB23 and ibrutinib suppresses the expression of c-Myc and E2F1 via YTHDF2-induced decay of mRNA.

(A-B) The relative m^{6}A enrichment of c-Myc (A) and E2F1 (B) mRNA in BT-549 cells treated with FB23, ibrutinib, or the combination of FB23 and ibrutinib, as well as the control group, for 24 h. m^{6}A levels were measured using m^{6}A-RIP-qPCR.

(C-D) The stability of c-Myc (C) and E2F1 (D) mRNA in BT-549 cells was assessed following 24 h treatment with FB23, ibrutinib, or their combination, as well as a control group. After treatment, cells were incubated with Act-D for 0-4 h to evaluate mRNA stability.

(E) The relative enrichment of c-Myc in YTHDF1, YTHDF2, and YTHDF3 were assessed in BT-549 cells pre-treated with FB23, ibrutinib, or their combination, as well as the control group, for 24 h. Enrichment was analyzed using RIP-qPCR.

(F) The relative enrichment of c-Myc in YTHDF1, YTHDF2, and YTHDF3 were assessed in BT-549 cells pre-treated with FB23, ibrutinib, or their combination, as well as the control group, for 24 h. Enrichment was analyzed using RIP-qPCR.

(**G**) Confirmation of YTHDF2 knockdown (KD) in MDA-MB-231 and BT-549 cells at the mRNA level, assessed using RT-qPCR.

(H-I) Effect of YTHDF2 knockdown on the mRNA stability of c-Myc (H) and E2F1 (I) in BT-549 cells treated with FB23 plus ibrutinib for 24 h and subsequently incubated with Act-D for 0-4 h.

The indicated *P* values (**$P < 0.01$, ****$P < 0.0001$) in panels A and B were determined using Student's t-test. For panels E, F, and G, *P* values (***$P < 0.0001$) were determined using one-way ANOVA followed by Dunnett's post hoc multiple comparisons test.

Table S1. The classification of targeted oncology drugs

**Target category**	**Drugs**	**Target**
Targeted protein tyrosine kinase	Ibrutinib	BTK; Src; Tyrosine kinases
	Nilotinib	Bcr-Abl
	Dasatinib	Bcr-Abl; c-Kit; Src
	Panotinib	Bcr-Abl
	Elortinib	EGFR
	Gefitinib	EGFR; Tyrosine kinases
	Dacomitinib	EGFR
	Crizotinib	ALK; c-Met/HGFR
	LDK37	ALK; IGF-1R; Serine protease
	Alectinib	ALK; Tyrosine kinases; VEGFR
	Lorlatinib	ALK; ROS; Tyrosine kinases
	Vandetanib	EGFR; VEGFR
	Lenvatinib	FGFR; PDGFR; VEGFR
	Axitinib	PDGFR; VEGFR; c-Kit
	Cabozantinib	c-Kit; c-Met/HGFR; TAM receptor; VEGFR; c-RET; FLT
	Regorafenib	c-Kit; c-RET; RAF; VEGFR

	Pazopanib	c-Kit; PDGFR; VEGFR
Targeted cell cycle protein	Ribociclib	CDK; VEGFR
	Abemaciclib	CDK
	Palbociclib	CDK
Targeted intracellular signaling pathway in inhibitor molecules	Encorafenib	RAF
	Binimetinib	MEK
	Cobimetinib	MEK
	Trametinib	MEK
Targeted DNA damage repair system	Niraparib	PARP; Others
Other targets	Ivosidenib	Isocitrate dehydrogenase (IDH)
	Embelin	IAP; Lipoxygenase; Prostaglandin receptor

Table S2. The primary and secondary antibodies

Antibodies	Source	Identifier
Anti-FTO antibody	Abcam	Cat# ab126605
Anti-c-Myc antibody [Y69]	Abcam	Cat# ab32072
Anti-E2F1 antibody	Abcam	Cat# ab288369
Anti-caspase3 antibody	Cell Signaling Technology	Cat# 9662
Anti-p53 antibody	Affinity Bioscience	Cat# AF0879
Anti-Bcl2 antibody	Abclonal	Cat# A20777
Anti-YTHDF1 antibody	Proteintech	Cat# 17479-1-AP
Anti-YTHDF2 antibody	Proteintech	Cat# 24744-1-AP
Anti-YTHDF3 antibody	Proteintech	Cat# 25537-1-AP
m^6A antibody	Synaptic Systems	Cat# 202003
IgG	Bioworld	Cat# B00051
GAPDH antibody (0411)	Santa Cruz	Cat# sc-47724
Goat anti-mouse IgG-HRP	Santa Cruz	Cat# sc-2354
Goat anti-rabbit IgG-HRP	Santa Cruz	Cat# sc-2004

Table S3 Primers for PCR assay.

Gene	Primer sequence	
c-Myc	Forward:	GTCAAGAGGCGAACACACAAC
	Reverse:	TTGGACGGACAGGATGTATGC
E2F1	Forward:	CATCCCAGGAGGTCACTTCTG
	Reverse:	GACAACAGCGGTTCTTGCTC
CDK4	Forward:	ATGGCTACCTCTCGATATGAGC
	Reverse:	CATTGGGGACTCTCACACTCT
BCL2L12	Forward:	CATGCTGGGAGCGTCACAT
	Reverse:	CTCCACTGAACTCGTACAAACTT
ATF4	Forward:	ATGACCGAAATGAGCTTCCTG
	Reverse:	GCTGGAGAACCCATGAGGT
HMGA1	Forward:	GCTGGTAGGGAGTCAGAAGGA
	Reverse:	TGGTGGTTTTCCGGGTCTTG
PABPC1	Forward:	CAGGCTCACCTCACTAACCAG
	Reverse:	GGTAGGGGTTGATTACAGGGT
GAPDH	Forward:	ACAACTTTGGTATCGTGGAAGG
	Reverse:	GCCATCACGCCACAGTTTC
YTHDF2	Forward:	CCTTAGGTGGAGCCATGATTG
	Reverse:	TCTGTGCTACCCAACTTCAGT
FTO	Forward:	CCAGAACCTGAGGAGAGAATGG
	Reverse:	CGATGTCTGTGAGGTCAAACGG
shFTO#1 Target & Flanking Sequence: CCCATTAGGTGCCCATATTTA		
shFTO#2 Target & Flanking Sequence: GCCAGTGAAAGGGTCTAATAT		
shFTO#3 Target & Flanking Sequence: TCGCATGGCAGCAAGCTAAAT		
YTHDF2-siRNA-l-sense GACCAAGAATGGCATTGCA		
YTHDF2 -siRNA-2-sense GCACAGAAGTTGCAAGCAA		

1. Materials and methods

1.1. shRNA and RNA and plasmid transfection

For shRNA silencing of FTO, we transfected lentiviruses expressing shRNAs against FTO#1, FTO#2, and FTO#3 into HEK 293T cells using Lipofectamine 2000 (Invitrogen, USA). After 48 h, we collected and concentrated the viruses. Subsequently, when the tumor cells reached 50%-60% confluence, we infected them with the concentrated virus. Finally, we selected the infected cells using Puromycin (3 μg/mL) for 3 days. The shRNA sequences are listed in Table S2.

Plasmid clones expressing c-Myc (OE-c-Myc), E2F1 (OE-E2F1), or an empty vector were purchased from Sino Biological Inc. (Beijing, China). Small interfering RNAs (siRNAs) designed for YTHDF2 (si-YTHDF2-1 and si-YTHDF2-2), and a negative control siRNA (si-NC) were obtained from RiboBio Co., Ltd. (Guangzhou, China). Transfection was conducted using Lipofectamine 2000 according to the manufacturer's instructions.

1.2. RNA extraction and real-time qPCR

RNA extraction and qRT-PCR procedures were outlined in our previous study [1], In brief, cultured cell samples were obtained, and following drug treatment, they were lysed in TRIzol reagent as per the manufacturer's instructions. The RNA yield and purity were assessed using NanoDrop 2000 (Thermo Fisher), and cDNA was subsequently synthesized using PrimeScript RT Master Mix (Takara, China). Real-time qPCR was conducted using SYBR Green PCR Master Mix (Takara) on a CFX96 Touch real-time System (Bio-Rad, USA). The specific genes examined in this study are listed in Table S2, with GAPDH serving as a normalization control. Relative gene expression levels were determined using the $2^{-\Delta\Delta CT}$ method.

1.3. m^{6}A-RNA immunoprecipitation qPCR (m^{6}A-RIP-qPCR)

Total RNA was extracted and quantified. IgG and m^{6}A antibodies were each incubated with Protein G beads (Invitrogen) at 4°C for 3 h. RNA input was collected prior to immunoprecipitation. For RIP, 100 μg of RNA was incubated with antibody-bound beads in reaction buffer (composed of 150 mM NaCl, 10 mM Tris-HCl at pH 7.5, and 0.1% NP-40 in nuclease-free H_2O) at 4°C for 3 h. Beads were sequentially washed with reaction buffer, low-salt buffer (10 mM Tris-HCl, pH 7.5, 50 mM NaCl, 0.1% NP-40), and high-salt buffer (10 mM Tris-HCl, pH 7.5, 500 mM NaCl, 0.1% NP-40). Bound RNA was eluted using Trizol and analyzed by RT-qPCR. m^{6}A enrichment of target RNAs was normalized to the IgG control.

1.4. Sample collection and preparation: RNA Quantification and Qualification

The quantification and integrity assessment of RNA were conducted employing the RNA Nano 6000 Assay Kit within the framework of the Bioanalyzer 2100 system (Agilent Technologies, USA). This initial phase underscores the meticulous approach required in handling RNA samples to ensure their suitability for subsequent analyses.

1.5. Library Preparation for Transcriptome Sequencing

In the preparatory phase for transcriptome sequencing, total RNA was designated as the foundational input material. The process commenced with the isolation of mRNA from the total RNA, utilizing poly-T oligo-attached magnetic beads. This step was critical for enriching mRNA concentrations, thereby enhancing the fidelity of subsequent sequencing efforts. Fragmentation of mRNA was achieved through the application of divalent cations at elevated temperatures within the First Strand Synthesis Reaction Buffer (5×), a crucial step for ensuring the generation of cDNA of optimal length for sequencing. The synthesis of the first strand cDNA was catalyzed using random hexamer primers and M-MuLV Reverse Transcriptase, followed by the strategic application of RNaseH to eliminate RNA strands, thus preventing RNA-DNA duplex formation. The synthesis of the second strand cDNA was facilitated by DNA Polymerase I and dNTP, with exonuclease/polymerase activities subsequently employed to refine the overhangs into blunt ends. This precision in cDNA preparation underscores the intricate balance between enzymatic activities required for high-quality library construction. Adaptors, characterized by their hairpin loop structures, were then ligated to the blunt-ended cDNA fragments. This step is pivotal for the hybridization process, enabling the selective amplification of target sequences during PCR. The specificity of cDNA fragments, aimed to be within the 370-420 bp range, was assured through purification with the AMPure XP system (Beckman Coulter, USA), illustrating the critical role of size selection in library quality. Following PCR amplification, the products underwent purification using AMPure XP beads, culminating in the final library. The rigorous purification process is indicative of the high standards maintained throughout the library preparation phase. Quality assurance of the library entailed a multi-faceted evaluation approach. Initial quantification was performed using a Qubit 2.0 Fluorometer, followed by dilution to an optimal concentration of 1.5 ng/μL. The Agilent 2100 Bioanalyzer played an instrumental role in determining the insert size, ensuring it met predefined criteria. The final quality confirmation was achieved through qRT-PCR, meticulously quantifying the library's effective concentration to exceed 2 nM. This layered approach to quality assessment underscores the comprehensive measures undertaken to ensure the library's integrity and suitability for high-throughput sequencing.

1.6. Gene set enrichment analysis (GSEA)

For GSEA, standard procedures as described by the GSEA user guide (http://www.broadinstitute.org/gsea/doc/GSEAUserGuideFrame.html) were used [2, 3].

1.7. Immunohistochemistry (IHC)

Immunohistochemistry was conducted to assess the expression levels of the target protein, following the methodology outlined in our prior research [4]. Images were photographed with a microscope.

References for supplementary data

1. Xie G, Wu X, Ling Y, Rui Y, Wu D, Zhou J, et al. A novel inhibitor of N^6methyladenosine demethylase FTO induces mRNA methylation and shows anti-cancer activities. Acta Pharm Sin B. 2022; 12: 853-66.
2. Subramanian A, Tamayo P, Mootha VK, Mukherjee S, Ebert BL, Gillette MA, et al. Gene set enrichment analysis: a knowledge-based approach for interpreting genome-wide expression profiles. Proc Natl Acad Sci U S A. 2005; 102: 15545-50.
3. Mootha VK, Lindgren CM, Eriksson K-F, Subramanian A, Sihag S, Lehar J, et al. PGC-1α-responsive genes involved in oxidative phosphorylation are coordinately downregulated in human diabetes. Nat Genet. 2003; 34: 267-73.
4. Chen Z, Wei W, Jiang G, Liu H, Wei W, Yang X, et al. Activation of GPER suppresses epithelial mesenchymal transition of triple negative breast cancer cells via NF-κB signals. Mol Oncol. 2016; 10: 775-88.
